# Pet-keeping in early life reduces the risk of allergy in a dose-dependent fashion

**DOI:** 10.1371/journal.pone.0208472

**Published:** 2018-12-19

**Authors:** Bill Hesselmar, Anna Hicke-Roberts, Anna-Carin Lundell, Ingegerd Adlerberth, Anna Rudin, Robert Saalman, Göran Wennergren, Agnes E. Wold

**Affiliations:** 1 Department of Paediatrics, Institute of Clinical Sciences, Sahlgrenska Academy, University of Gothenburg, Gothenburg, Sweden; 2 Department of Rheumatology and Inflammation Research, Institute of Medicine, Sahlgrenska Academy, University of Gothenburg, Gothenburg, Sweden; 3 Department of Infectious Diseases, Institute of Biomedicine, Sahlgrenska Academy, University of Gothenburg, Gothenburg, Sweden; Université Paris Descartes, FRANCE

## Abstract

**Objectives:**

Several studies have indicated that early pet keeping could protect the infant from later allergy development. Here, we investigate if there is a dose-dependent association between cat- and dog-keeping during the first year of life and subsequent allergy development.

**Methods:**

Two cohorts were investigated: a cross-sectional questionnaire-based study of 7- to 8-year-old children (N = 1029) from Mölndal and Kiruna, and a birth-cohort of children from the Västra Götaland county clinically evaluated for asthma and allergy by paediatricians up to the age of 8–9 years (N = 249). The cross-sectional study asked validated questions on asthma and allergy that had been used in two previous studies of children from the same areas. In the birth-cohort study, a diagnosis of asthma and allergy was based on predefined clinical criteria, and laboratory evaluation included blood eosinophils, skin-prick tests and specific immunoglobulin E analyses. Information on pets during first year of life was collected retrospectively in the Cross-Sectional Cohort and prospectively in the Birth Cohort.

**Results:**

A dose-response association was seen, with less allergic manifestations (any of asthma, allergic rhinoconjunctivitis, or eczema) with increasing number of household cats and dogs during the first year of life. In the Cross-Sectional Cohort, allergy ever decreased from 49% in those with no pets to zero in those with five or more pets (P-value for trend 0.038), and from 32% to zero for allergy last year (P-value for trend 0.006). The same pattern was seen in Birth Cohort. Sensitization to animals, as well as pollens, also decreased with increasing number of animals in the household.

**Conclusion:**

The prevalence of allergic disease in children aged 7–9 years is reduced in a dose-dependent fashion with the number of household pets living with the child during their first year of life, suggesting a “mini-farm” effect, whereby cats and dogs protect against allergy development.

## Introduction

The clinical consequences of exposure to different allergens in early life have long been a matter of discussion, especially if infants are exposed to pets such as cats and dogs during their first year of life. Early pet-keeping was previously considered to be a risk factor for allergy development, but several studies from the last 20 years have highlighted that this is probably not the case [1–8], even in individuals with a strong family history of atopy [9]. Today, early pet-keeping is generally not considered to be a risk factor for allergy in families with otherwise healthy infants.

Conversely, pet-keeping during early life may instead protect from later allergy [1], especially exposure to more than one dog or to both a cat and a dog [3, 4]. We were the first to demonstrate, in 1999 [1], that children in families keeping (a) cat(s) or (a) dog(s) during the child´s first year of life had less asthma at 7–9 years as compared to children with no such animals, and that this difference remained also after adjusting for selection mechanisms due to allergy among parents or siblings. The existence of an allergy-protective effect from pet-keeping is also supported by immunological data. In studies analysing the effect of cat exposure on asthma and allergy development, a high-dose exposure to cat allergens [10], or keeping of cats [11], were associated with clinical tolerance and cat-specific IgG4, but not IgE.

Immunological tolerance facilitated by keeping of cats and dogs during early life is, however, still a hypothesis, despite some support for this assumption in the aforementioned studies. Not all studies report a long-term protective effect [7], and if such an effect exists, it is still not known how induction of this immunological tolerance is mediated. In principle, we hypothesized that two different mechanisms–not mutually exclusive–could contribute to a protective effect of pet-keeping. First, exposure to cat or dog dander, containing massive amounts of allergens from the respective species, could induce high-dose clinical tolerance to the allergens, i.e. reduced risk of cat-allergy in the children exposed to cats and dog-allergy in children with dogs. Second, cohabiting pet animals could provide a “mini-farm” environment, with microbes or other immunoregulatory factors that provide a broad modifying effect on immune development in the child, leading to tolerance not only to the pet itself, but also to food and airborne allergens. In this study we try to address this question, hypothesising that high-dose allergen exposure should induce tolerance only to that specific type of animal, whereas a mini-farm induced tolerance is supposed to be protective not only to a specific animal but also to other environmental allergens.

Most often research focus on identifying risk factors for allergy development. But in modern society, finding lifestyle factors that could protect from allergy has become equally important. The main aim of this study was to investigate if pet-keeping during early life affects later allergy development and, if so, whether a dose-response association was detectable. Second, if the protective effect was species-specific, suggesting an allergen-driven tolerance induction, or, if it is species-unspecific suggesting an allergy-protective “mini-farm” environment. We used data from a cross-sectional cohort and a birth cohort for the analyses to minimize influences from common methodological shortcomings, e.g. selection bias and reverse causation.

## Methods

The analysis was based on two study populations. A cross-sectional questionnaire study was performed in 2007 in 7–8 year old children (Cross-Sectional Cohort, N = 1029). The other study population was the Birth Cohort, recruited between 1998 and 2007.

### Cross-Sectional Cohort

In the cross-sectional cohort, a questionnaire on asthma and allergy was distributed to all 7- to 8-year-old children in Mölndal, a small town which is part of the Gothenburg urban area on the South West Sweden, and Kiruna, a town in the far north of Sweden. Of 1838 questionnaires distributed, 1029 (56%) were returned. We used the same questions on asthma, eczema, and allergic rhinoconjunctivitis (ARC) as had been used in two previous studies of children from the same regions and of similar ages in 1979 and in 1991 [12, 13]. Diagnostic criteria and information gathered on pet exposure are shown in Table 1.

**Table 1:** Diagnostic criteria and information on pet exposure in the Cross-Sectional Cohort and Birth Cohort.

Criterion	Cross-Sectional Cohort	Birth Cohort
Diagnosis of asthma	“Asthma ever” diagnosed if there was a positive response to: “Has your child had asthma or asthmatic bronchitis”? “Current asthma” diagnosed if there was a positive response to “Has your child had asthma or asthmatic bronchitis in the previous year”?	Asthma diagnosed at age 8–9 years if the child in the last 12 months had symptoms of wheeze/heavy breathing together with: FEV_1_ reversibility >12%, or bronchial hyperresponsiveness to methacholine (PD_20_ <0.6 mg), or ongoing controller medication with inhaled corticosteroids or leukotriene antagonist
Diagnosis of ARC	ARC diagnosed if there was a positive response to: “Has your child had allergic rhinitis or allergic conjunctivitis”? “Current ARC” diagnosed if there was a positive response to: “Has your child had allergic rhinitis or allergic conjunctivitis in the previous year”?	ARC diagnosed at age 8–9 years if the child in the last 12 months had eye or nose symptoms suggestive of allergic disease together with a positive skin-prick test or specific IgE to the relevant allergen
Diagnosis of eczema	“Eczema ever” diagnosed if there was a positive response to: “Did your child ever have eczema”? “Current eczema” diagnosed if there was a positive response to: “Did your child have eczema in the previous year”?	Eczema diagnosed at age 8–9 years if the child in the last 12 months had a skin condition fulfilling Williams criteria [16], or an itching dermatitis that had been chronic or relapsing for ≥6 months
Diagnosis of allergy	“Allergy ever” and “allergy last year” included any of asthma, ARC, or eczema ever or last year, respectively	“Allergy last year” included any of asthma, ARC, or eczema
Cats and dogs in household	Number of cats and dogs in the household during the first year of life	Data on number of cats and dogs was obtained from the 6-month telephone interview

ARC, allergic rhinoconjunctivitis; FEV_1_, forced expiratory volume in 1 s; Ig, immunoglobulin; PD_20_: provocative dose inducing a fall of ≥20% in FEV_1_.

### Birth Cohort

The Birth-Cohort was pooled data from two birth-cohorts in the Västra Götaland county. Between 1998 and 2003, 184 children in the ALLERGYFLORA were recruited from Mölndal in the Gothenburg urban area [14]. The ALLERGYFLORA was designed to analyse the effects of early life events and early gut colonisation on later allergy development. The second group was the FARMFLORA. The study is a copy of the ALLERGYFLORA, but the children are living in a rural region. Children were recruited between 2005 and 2007, from a farming area in Skaraborg, northeast of Gothenburg, comprising 28 children living on dairy farms and 37 children living in the same rural area but not on farms [15]. The parents were all contacted before the birth, and children born ≥38 gestational weeks were included in the study on day 0–3 after delivery. The parents were interviewed when their children were aged 6 and 12 months; clinical examinations by paediatric allergologists were done at age 18 months and 3 and 8–9 years. Diagnostic criteria and information gathered on pet exposure are detailed in Table 1.

### Lung function tests

Lung function tests were done in the Birth Cohort. Before lung function tests, children were not permitted: tea, coffee, or cola drinks within 4 hours; short-acting beta-agonists within 8 hours; ipratropium bromide within 24 hours; long-acting beta-agonists, theophylline, or nasal steroids within 48 hours; or antihistamines within 72 hours to 1 week, depending on the type of drug. Methacholine challenges were not performed during the pollen season in pollen-allergic children; if the child had a viral infection or common cold within 14 days; if oral steroids had been given within 14 days; if forced expiratory volume in 1 s (FEV_1_) was <65% predicted; or if the child had a heart disorder. All lung function tests were done in a sitting position and a nose clamp was used.

Flow-volume curves and reversibility tests were performed in accordance with American Thoracic Society and European Respiratory Society guidelines [17] using Spida 5 spirometry software (Micro Medical Limited, Rochester, UK). A bronchodilator response was considered positive if FEV_1_ increased by >12% from baseline [18].

Airway hyperresponsiveness was determined by direct methacholine challenge [19], using a tidal volume-triggered dosimetric method (Spira Elektro 2 jet nebulizer; Spira Respiratory Care Centre Ltd, Hämeenlinna, Finland). Basic FEV_1_ was determined after inhalation of isotonic saline. Methacholine was subsequently inhaled in increasing doses at intervals of at least 1 minute until FEV_1_ had decreased by ≥20%, or a cumulative dose of 6.1875 mg had been given. At the end of the challenge, all subjects received an inhalation of salbutamol and FEV_1_ was measured to ensure recovery (FEV_1_ >90% of baseline value). The provocative dose inducing a fall of ≥20% in FEV_1_ (PD_20_) was determined by interpolating the dose-response curve; airway hyperresponsiveness was defined as PD_20_ <0.6 mg. The slope was calculated from the maximum fall in FEV_1_ divided by the cumulative dose.

### Eosinophils, specific immunoglobulin E, and Skin-prick tests

Blood tests and Skin-Prick Tests were done in the Birth Cohort. Blood eosinophil cells, specific immunoglobulin E (IgE), and total IgE were all analyzed at the Sahlgrenska University Hospital. All analyses were accredited by the Swedish Board for Accreditation and Conformity Assessment. For specific IgE and total IgE, Phadiatop and ImmunoCAP tests were used (Thermo Fisher Scientific, Uppsala, Sweden). Skin-prick tests (SPTs) were carried out for common airborne allergens (cat, dog, horse, rabbit, birch, grass, mugwort, *Dermatophagoides pteronyssinus*, *Dermatophagoides farinae*, and *Cladosporium herbarum*) according to the standards of the Subcommittee on Skin Tests of the European Academy of Allergy and Clinical Immunology [20]. Allergen extracts were all manufactured by ALK (Hørsholm, Denmark). A positive SPT corresponds to a weal with a diameter exceeding the negative control by ≥3 mm.

### Statistical analysis

Analyses were performed with SPSS statistical software (version 24; IBM Corp., Armonk, NY, USA); for the multivariate analyses we used SIMCA-P+ software (version 14.1; MKS Umetrics AB, Umeå, Sweden).

χ^2^ tests were used to compare differences between proportions. Trend analyses were based on linear-by-linear association and exact tests. Backward logistic regression models were used to control for covariates and possible confounders. A two-sided *P*-value <0.05 was considered statistically significant.

Orthogonal projection to latent structures (OPLS), an extension of PLS-regression (Partial Least Square regression) in order to improve interpretability, was used in the birth-cohort study to analyse the relationship between the number of pets at 6 months of age, parental history of allergy, and 12 independent outcomes from the follow-up at 8–9 years. B coefficients on scaled and centered data were calculated with 95% confidence intervals.

### Ethics

Written informed consent was obtained from all parents. The study was approved by the Ethics Committee of the University of Gothenburg, Sweden (R448-97 and Ö 446–00) and the Human Research Ethics Committee of the Medical Faculty, University of Gothenburg, Sweden (Dnr. 321–05, 363–05, 105–07 and 674–14).

## Results

In both the Cross-Sectional Cohort and the Birth Cohort, the sex ratios were 50:50 or close to it (Table 2). A parental history of allergy was slightly less common in the Birth Cohort, probably due to the stricter diagnostic criteria used requiring a doctor’s diagnosis of allergic disease. In children, the prevalence of allergic disease (allergy last year) was similar in the Cross-Sectional Cohort and the Birth Cohort.

**Table 2:** Characteristics of the study populations.

	Cross-Sectional Cohort (n = 1029)	Birth Cohort (n = 249)
Boys, n (%)	483 (47)	125 (50)
History of allergy^a^, n (%)		
Mother	498 (48)	110 (44)
Father	399 (39)	90 (36)
Parent with a university degree, n (%)		
Mother	321 (31)	–
Father	277 (28)	–
Number of pets		
0	767	181
1	165	40
2	64	–
≥2	–	28
3	21	–
4	7	–
≥5	2	–
Children with allergy, n (%)		
Ever	481 (47)	95 (38)
In the last year	314 (31)	73 (29)

^a^In the cross-sectional study: A history of asthma or allergic rhinoconjunctivitis ever. In the birth-cohort study, a doctor’s diagnosis of asthma, allergic rhinoconjunctivitis, or eczema.

In the Cross-Sectional Cohort, allergy was based on a history of asthma, ARC, or eczema (allergy ever), or, asthma, ARC, or eczema with symptoms in the last 12 months (allergy last year). In the Birth Cohort, allergy ever was based on a diagnosis of asthma, ARC, or eczema at any of the follow-ups (18 months, 3 years, or 8–9 years), and allergy last year was based on current asthma, ARC, or eczema with symptoms in the 12-month period preceding the follow-up at age 8–9 years.

The number of household dogs and cats during first year of life was set to range from zero to ≥5 in the Cross-Sectional Cohort; in the smaller Birth Cohort, the number of pets at 6 months of age was recorded in a range from zero to ≥2.

**Fig 1:**
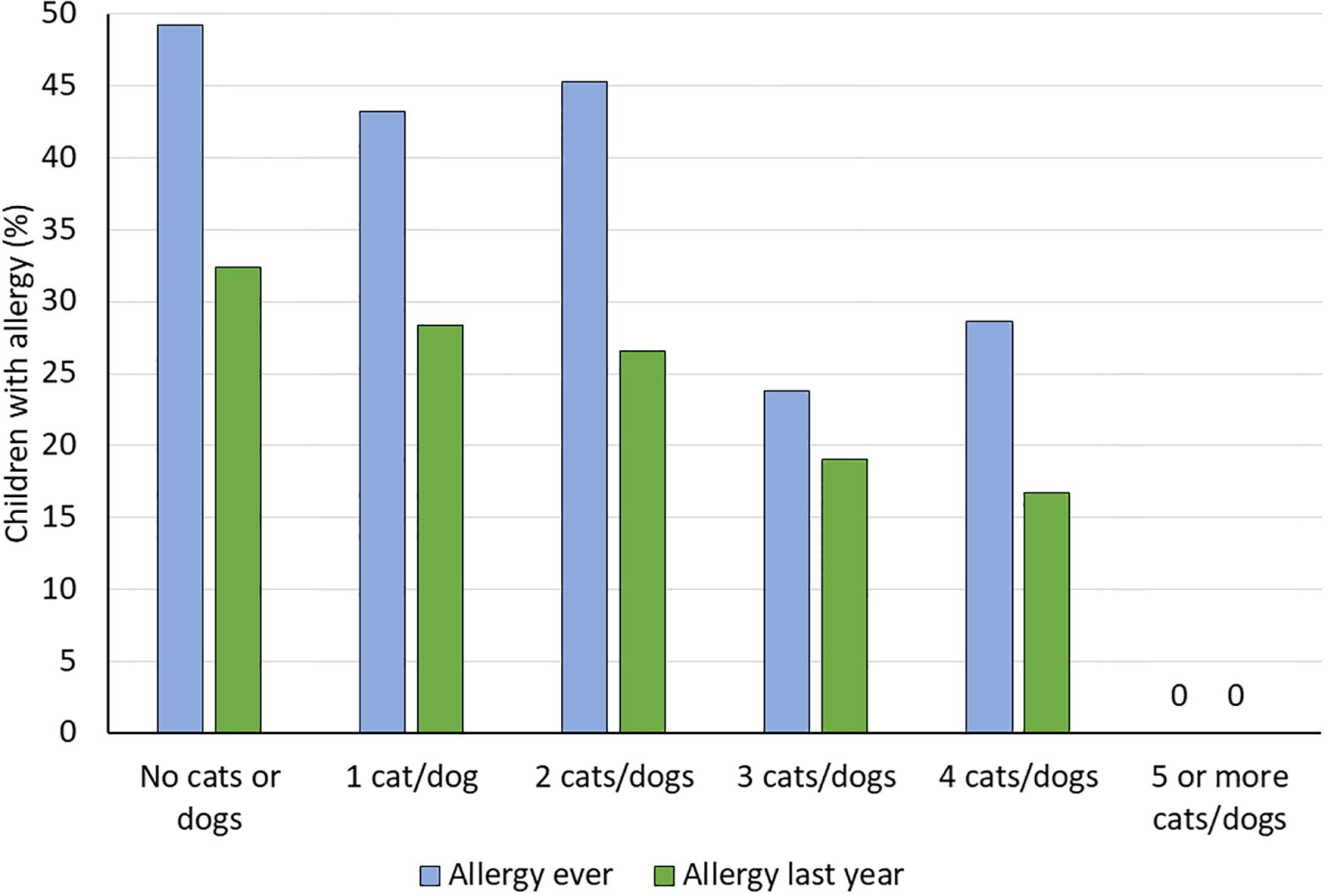
Data from the cross-sectional study. Allergy (any of asthma, allergic rhinoconjunctivitis, or eczema) in relation to the number of household cats and dogs during the child’s first year of life. Allergy last year required current symptoms, i.e. symptoms in the last 12 months.

Fig 1 shows the cumulative incidence (allergy ever) and prevalence (allergy last year) of allergic disease in relation to the number of household cats and dogs during the first year of life for the Cross-Sectional Cohort. Both allergy ever and allergy last year decreased with increasing number of cats and dogs (*P*-value for trend with exact test: 0.006 for allergy ever and 0.038 for allergy last year).

**Fig 2:**
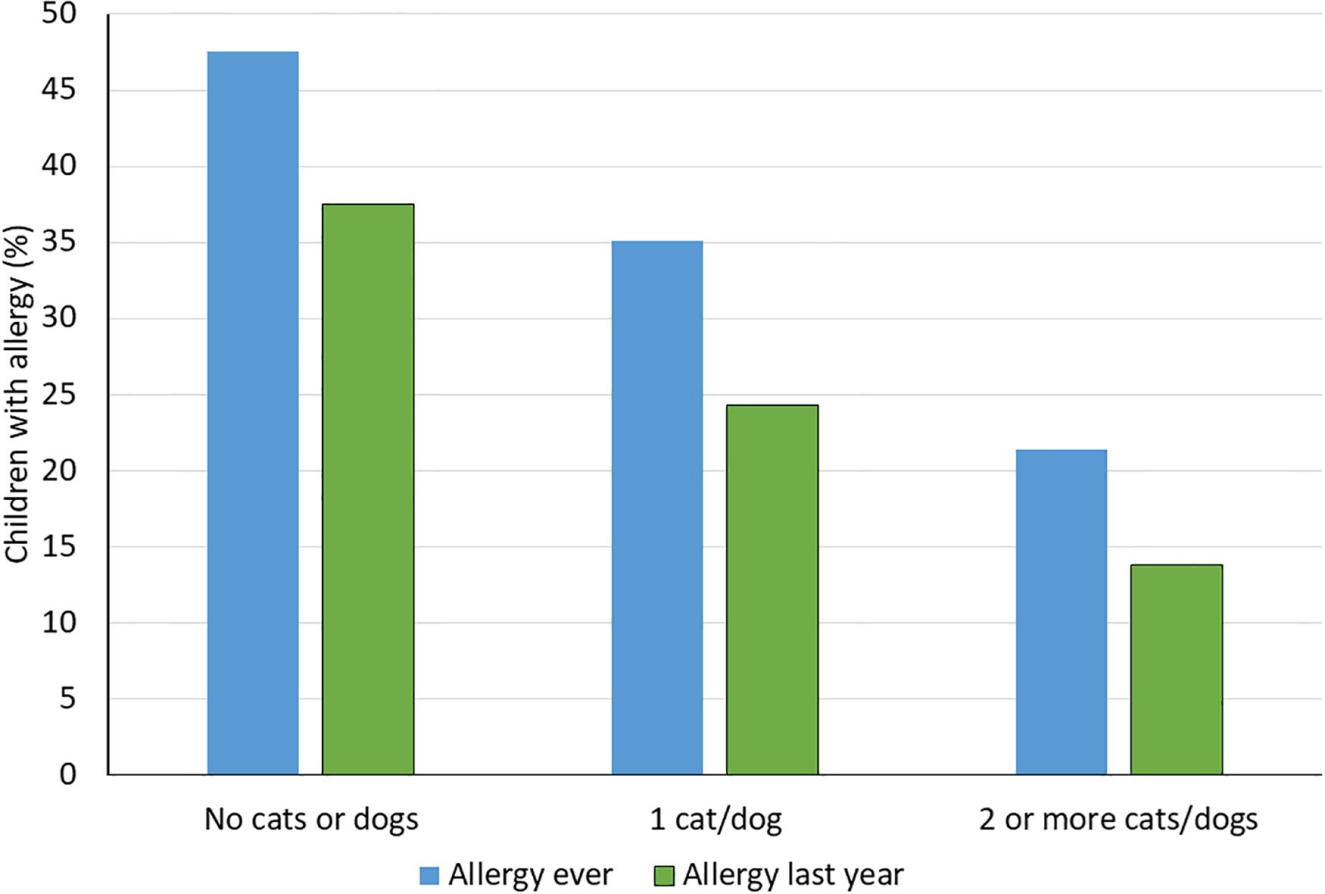
Data from the Birth Cohort. Allergy (any of asthma, allergic rhinoconjunctivitis, or eczema) in relation to the number of household cats and dogs when the child was 6 months old. Allergy last year required current symptoms, i.e. symptoms in the last 12 months.

A similar pattern was seen in the Birth Cohort, with a decreasing frequency of allergic disease (both current and ever) with increasing number of household cats and dogs (Fig 2; *P*-value for trend: 0.007 for allergy ever and 0.008 for allergy last year).

Backward multiple logistic regression analyses, with allergy ever as independent variable, were used on both the Cross-Sectional and Birth Cohort. In the Cross-Sectional Cohort, independent variables were sex, parental history of allergy, number of siblings, and number of pets during first year of life. In the final step, only pets and parental history of allergy remained, giving an odds ratio of 0.80 for every additional animal (*P* = 0.012). In the Birth Cohort, the same independent variables were included. In the final step, only pets during first year of life and parental history of allergy remained, giving an odds ratio of 0.65 for each additional animal (*P* = 0.058).

To further analyse a possible influence of parental allergic disease on the families’ choice to have pets, parental sensitization was analysed in relation to number of household pets. In the first 184 Birth Cohort-children from the Gothenburg-Mölndal area, parents were tested for sensitization with the Phadiatop test. Blood samples were obtained from 149 mothers and 141 fathers. There was no statistically significant difference in the frequency of positive Phadiatop tests from parents with no household pets when their child was 6 months old versus parents with increasing number of animals (Table 3).

**Table 3:** Sensitisation in parents, measured with Phadiatop tests, in relation to the number of household cats and dogs the family had when their child was 6 months old.

Number of cats or dogs when the child was 6 months old	Positive Phadiatop test result, n/N (%)	
	Mother (n = 149)	Father (n = 141)
0	65/127 (51)	73/121 (60)
1	9/18 (50)	7/17 (41)
≥2	3/4 (75)	2/3 (67)
*P*-value for trend^a^	0.590	0.425

^a^Based on exact tests

**Fig 3:**
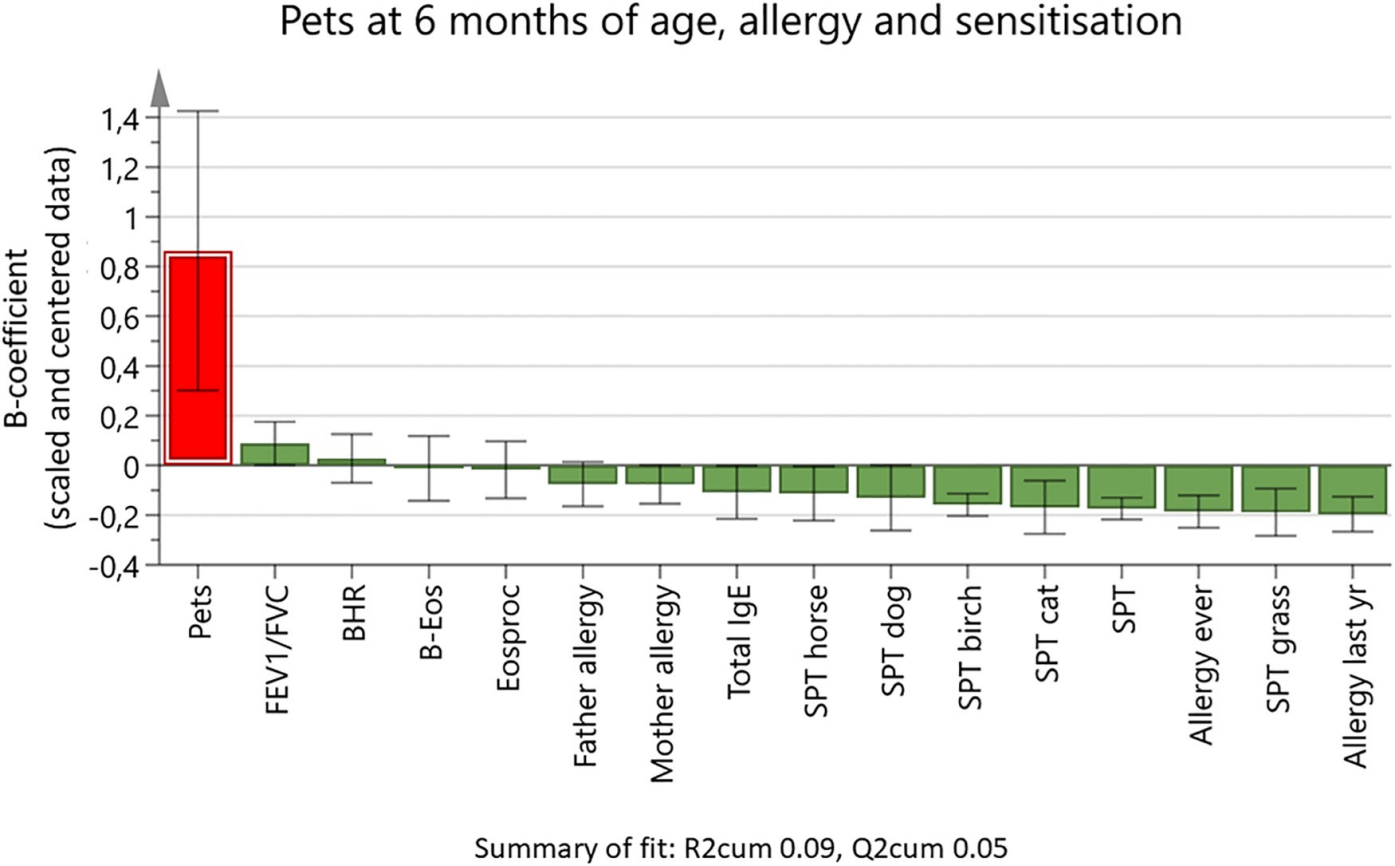
Orthogonal projection to latent structures loading plot showing associations between the number of household cats and dogs when the child was 6 months old (Y variable), and a set of 15 X variables. The outcome variables for lung function (forced expiratory volume in 1 s [FEV_1_]/forced vital capacity [FVC]), bronchial hyperresponsiveness (BHR), blood eosinophil count (B-Eos), percentage of blood eosinophils (Eosproc), total immunoglobulin (IgE), and skin-prick tests (SPTs) were from the age 8–9 years follow-up. SPTs are given as weal diameter. X variable bars pointing in the same direction as the Y variable are positively associated with the Y variable, and bars pointing in the opposite direction are negatively associated. The height of the bars shows the B-coefficients for scaled and centered data, with 95% confidence intervals.

In the Birth Cohort, the relationship between the number of household pets at 6 months old and sensitisation at 8–9 years old was tested in an OPLS analysis (Fig 3), a regression model suited to test how a large set of X-variables relate to Y-variable(s). The number of pets was used as the Y variable (the left bar). The figure shows how the other variables (X variables) are related to the Y variable. X-variable bars in the same direction as the Y variable bar are positively associated; bars pointing in the opposite direction to the Y-variable bar are negatively associated. The main finding was that the degree of sensitization in children, expressed as SPT diameter, decreased with increasing number of pets, and that this association was seen not only for sensitization to pets but also for sensitization to pollen (birch and grass). No significant association was found between number of pets and presence of allergy in mother or father.

## Discussion

In 1999 we published a study showing that early pet-keeping was associated with less allergy development in children [1]. Since then, several studies have been published supporting our finding [2–6, 8], but others did not [7]. A common interpretation of published data has, to date, been that early keeping of cats or dogs does not increase the risk of allergy but we do not definitely know if it has any protective effect, in the same way as farm animals and farm-living have [21–23]. However, the main findings from this extended study support our previous results, that pet-keeping during early childhood is associated with less allergy, and that the protective effect from pet-keeping increased with increasing number of animals. Furthermore, the protective effect influenced not only clinical allergy but also sensitization to both animals and pollen, suggesting an underlying “mini-farm” mechanism.

In this study we used results from two different study populations in order to address common questions raised when interpreting results from studies investigating allergy prevention. The first, and most obvious question is if the findings could be due to a type-1 error, i.e. a false positive. As we found similar results in both populations studied, we argue that our main finding was not due to a type-1 error. Epidemiological cross-sectional studies have several advantages as they usually include a large population, making them suitable for both univariate and multivariate analyses, but other biases and diagnostic validity are always issues to be considered. We used the Cross-Sectional Cohort for the main analyses, and these were then repeated in the Birth Cohort. As the results from the Cross-Sectional Cohort were reproduced in the Birth Cohort, we argue that neither recall bias nor reverse causation explain the results. Neither was diagnostic validity a major problem in the Cross-Sectional Cohort, as the main finding was repeated in the Birth Cohort in which strict diagnostic criteria were used. A similar argument could also be used when assessing parental history of allergy. In the Cross-Sectional Cohort, a healthy pet-owner effect might be an issue, but in the Birth Cohort the information on parental allergy was collected when the child was just a few days old. Furthermore, parental sensitization data from the Birth Cohort does not indicate any major difference in sensitization pattern between parents with versus without pets. Selection bias is another issue often discussed for this type of study, as allergic parents are not supposed to own cats or dogs. One way to handle this issue is to ask parents about their reasons for not owing pets, as we did in our 1999 study [1]. Another method is to conduct a dose-response analysis, as we have done in this study. The rationale behind this approach is that selection may occur between families having versus not having animals, rather than between having one or two animals versus two or three animals. To summarise, it is our view that selection bias, recall bias, reverse causation, or imprecise diagnostic validity do not explain our finding of an inverse correlation between the number of household cats and dogs during a child’s first year of life and allergy prevalence.

The dose-response effect and a similar protective effect for sensitisation to animals and pollen, indicate that the protective effect is mediated by the keeping of animals, and is not a species-specific effect. It is our suggestion that the allergy-protective effect mediated by pet-keeping should be considered as a “mini-farm” effect, equating our findings to those found in the numerous farm studies performed [24]. A “mini-farm” effect could also explain why a protective effect is found in some studies, but not all. The effectiveness of an unspecific allergy-preventive (or immune-stimulating) agent should be seen in the light of other protective factors. This was demonstrated elegantly by Matricardi et al, who showed that siblings only had an allergy-protective effect in subjects seronegative for hepatitis A and not in those who were seropositive, i.e. those who already had a strong allergy-protective effect from hepatitis A or an environment where hepatitis A is common [25]. A dog or a cat may thus have a protective effect in children who have few other protective factors, provided that the child has close contact with the animal during their early years. If the child already has several other protective factors, a dog or cat may not add any extra protection, unless the child is exposed to several animals, i.e. a “mini-farm”. Although several studies have shown that pet-keeping, mainly from direct exposure [26], in early life is associated with less asthma or allergy [2–6, 8, 26], especially if exposed to more than one animal [3, 4], such an effect is not found in all studies. In a large study with pooled data from several birth cohorts, neither a protective effect, nor an increased allergy risk from early pet-keeping was found [7]. However, not finding a protective effect in a study population does not necessarily mean that a protective effect does not exist during certain circumstances, as previously mentioned. Protection seems more likely if exposure occurs at close quarters, i.e. direct exposure [8], if other strong allergy-protective factors are missing, or, as in our study, if the child is exposed to more than one animal [3, 4]. Thus, it is plausible that tolerance induction via a mini-farm mechanism require a close contact with the animal(s), otherwise the child will only be exposed to allergens from the animal, not the microbes and endotoxins shred by the animal, components that seems to be important in tolerance induction. And close contact with the pet animal is probably more common in urban areas, where families use to keep their pet animals inside the house or flat. In rural areas, dogs and cats are more often kept outdoors. In such cases, allergens from cats and dogs will still be spread inside the house, causing sensitisation, but not the microbes and microbial products that follow a close contact with the animal. Cleaning habits may also affect the effectiveness of pet-induced tolerance induction. Allergens are seldom reduced by excessive cleaning [27], but the mini-farm environment might be less effective.

The mechanisms behind the proposed “mini-farm” effect from dogs and cats can, of course, only be speculated on, but according to the hygiene hypothesis [28], immune stimulation by microbial exposure might be one possible mechanism. We have found support in various studies for allergy protection by early microbial exposure [29, 30] or presumed early microbial exposure [31], and dogs and other pet animals seem to have this capability [8].

With the study design, we have been able to show a negative association between the number of animals in the child’s home during the first year of life and allergy development, but the study has limitations. The Cross-Sectional Cohort had a response rate of slightly less than 60%, which may select a more allergy-prone population, even though we have not found any indications for such a selection [32]. Furthermore, recall bias and diagnostic validity may be limitations in cross-sectional questionnaire studies, but these limitations are balanced by the concordant results found in the Birth Cohort. Similarly, the smaller Birth Cohort population is balanced by the much larger population size in the Cross-Sectional Cohort.

In conclusion, the prevalence of allergic disease in children aged 7–9 years is reduced in a dose-response pattern with increasing number of cats and dogs in the home during the first year of life, suggesting a “mini-farm” effect whereby pet-keeping protects against allergy development.

## Supporting information

S1 Table Cross-Sectional Cohort. (DOCX)

S2 Table Birth Cohort. (DOCX)

S3 Table Cross-Sectional Cohort logistic regression. (DOCX)

S4 Table Birth Cohort logistic regression. (DOCX)

S5 Table Birth Cohort OPLS. (DOCX)

S6 Table Birth Cohort parental sensitization. (DOCX)
