# Supplementary material for: Pet-keeping in early life reduces the risk of allergy in a dose-dependent fashion
Source: PLoS One. 2018 Dec 19;13(12):e0208472. doi: 10.1371/journal.pone.0208472 (PMC6300190; doi:10.1371/journal.pone.0208472)
Supplement: S1 Table — (DOCX) [file pone.0208472.s001.docx]

| Pets_1yr | Allergy_ever | Allergy_8yr |
| --- | --- | --- |
| 0 | 1 | 1 |
| 0 | 1 | 1 |
| 0 | 0 | 0 |
| 0 | 0 | 0 |
| 0 | 0 | 0 |
| 0 | 0 | 0 |
| 1 | 0 | 0 |
| 0 | 0 | 0 |
| 1 | 0 | 0 |
| 0 | 1 | 1 |
| 0 | 1 | 1 |
| 0 | 0 | 0 |
| 0 | 1 | 1 |
| 0 | 0 | 0 |
| 0 | 0 | 0 |
| 2 | 1 | 0 |
| 0 | 0 | 0 |
| 2 | 1 | 1 |
| 0 | 1 | 1 |
| 0 | 0 | 0 |
| 0 | 1 | 1 |
| 0 | 1 | 1 |
| 1 | 1 | 1 |
| 3 | 0 | 0 |
| 0 | 0 | 0 |
| 1 | 0 | 0 |
| 0 | 1 | 1 |
| 0 | 0 | 0 |
| 0 | 1 | 0 |
| 3 | 0 | 0 |
| 0 | 1 | 0 |
| 0 | 1 | 1 |
|  | 1 | 1 |
| 0 | 1 | 1 |
| 1 | 0 | 0 |
| 0 | 1 | 0 |
| 0 | 1 | 0 |
| 1 | 1 | 0 |
| 0 | 0 | 0 |
| 0 | 1 | 1 |
| 0 | 1 | 1 |
| 1 | 1 | 1 |
| 0 | 0 | 0 |
| 0 | 0 | 0 |
| 0 | 1 | 0 |
| 1 | 1 | 1 |
| 0 | 1 | 1 |
| 0 | 1 | 0 |
| 1 | 1 | 0 |
| 2 | 1 | 1 |
| 1 | 0 | 0 |
| 1 | 0 | 0 |
| 0 | 1 | 1 |
| 0 | 1 | 0 |
| 0 | 1 | 1 |
| 0 | 0 | 0 |
| 0 | 0 | 0 |
| 0 | 1 | 1 |
| 0 | 1 | 0 |
| 0 | 0 | 0 |
| 0 | 0 | 0 |
| 0 | 1 | 1 |
| 0 | 1 | 1 |
| 0 | 1 | 1 |
| 1 | 0 | 0 |
| 2 | 0 | 0 |
| 0 | 0 | 0 |
| 0 | 0 | 0 |
| 0 | 1 | 0 |
| 0 | 0 | 0 |
| 2 | 1 | 0 |
| 0 | 0 | 0 |
| 0 | 1 | 0 |
| 4 | 0 | 0 |
| 0 | 1 | 1 |
| 0 | 0 | 0 |
| 0 | 1 | 0 |
| 0 | 1 | 0 |
| 0 | 0 | 0 |
| 0 | 0 | 0 |
| 0 | 0 | 0 |
| 0 | 0 | 0 |
| 0 | 1 | 1 |
| 0 | 1 | 1 |
| 1 | 0 | 0 |
| 1 | 0 | 0 |
| 0 | 0 | 0 |
| 0 | 1 | 1 |
| 0 | 1 | 1 |
| 0 | 1 | 1 |
| 1 | 1 | 1 |
| 0 | 0 | 0 |
| 0 | 1 | 1 |
| 0 | 1 | 1 |
| 0 | 0 | 0 |
| 3 | 0 | 0 |
| 0 | 0 | 0 |
| 2 | 0 | 0 |
| 1 | 0 | 0 |
| 0 | 0 | 0 |
| 0 | 0 | 0 |
| 0 | 1 | 1 |
| 1 | 0 | 0 |
| 0 | 1 | 1 |
| 0 | 1 | 1 |
| 0 | 0 | 0 |
| 0 | 1 | 1 |
| 0 | 1 | 0 |
| 1 | 0 | 0 |
| 0 | 1 | 1 |
| 0 | 0 | 0 |
| 1 | 0 | 0 |
| 1 | 0 | 0 |
| 0 | 0 | 0 |
| 0 | 0 | 0 |
| 0 | 0 | 0 |
| 0 | 0 | 0 |
| 0 | 0 | 0 |
| 0 | 1 | 1 |
| 0 | 0 | 0 |
| 0 | 0 | 0 |
| 0 | 0 | 0 |
| 0 | 0 | 0 |
| 0 | 1 | 1 |
| 0 | 1 | 1 |
| 0 | 1 | 1 |
| 1 | 1 | 1 |
| 1 | 0 | 0 |
| 1 | 0 | 0 |
| 0 | 1 | 1 |
| 1 | 1 | 0 |
| 0 | 0 | 0 |
| 0 | 1 | 1 |
| 1 | 0 | 0 |
| 0 | 1 | 0 |
| 3 | 1 | 1 |
| 0 | 1 | 1 |
| 0 | 1 | 0 |
| 2 | 0 | 0 |
| 1 | 1 | 0 |
| 0 | 0 | 0 |
| 1 | 0 | 0 |
| 0 | 0 | 0 |
| 0 | 0 | 0 |
| 0 | 0 | 0 |
| 0 | 0 | 0 |
| 0 | 0 | 0 |
| 0 | 0 | 0 |
| 1 | 0 | 0 |
| 2 | 1 | 1 |
| 2 | 0 | 0 |
| 0 | 1 | 1 |
| 1 | 0 | 0 |
| 1 | 1 | 0 |
| 0 | 1 | 0 |
| 0 | 0 | 0 |
| 0 | 1 | 1 |
| 0 | 1 | 0 |
| 4 | 0 | 0 |
| 0 | 0 | 0 |
| 0 | 0 | 0 |
| 0 | 1 | 1 |
| 1 | 0 | 0 |
| 0 | 0 | 0 |
| 1 | 0 | 0 |
| 0 | 0 | 0 |
| 0 | 0 | 0 |
| 0 | 0 | 0 |
| 0 | 0 | 0 |
| 3 | 0 | 0 |
| 3 | 0 | 0 |
| 0 | 1 | 1 |
| 0 | 1 | 1 |
| 2 | 0 | 0 |
| 1 | 0 | 0 |
| 0 | 1 | 1 |
| 1 | 0 | 0 |
| 1 | 0 | 0 |
| 0 | 0 | 0 |
| 0 | 1 | 1 |
| 1 | 0 | 0 |
| 1 | 0 | 0 |
| 1 | 1 | 1 |
| 0 | 1 | 0 |
| 1 | 0 | 0 |
| 0 | 1 | 0 |
| 0 | 0 | 0 |
| 0 | 1 | 0 |
| 0 | 0 | 0 |
| 2 | 0 | 0 |
| 0 | 1 | 0 |
| 2 | 0 | 0 |
| 3 | 1 | 0 |
| 0 | 1 | 1 |
| 0 | 1 | 1 |
| 1 | 0 | 0 |
| 0 | 1 | 0 |
| 0 | 1 | 1 |
| 0 | 1 | 1 |
| 0 | 1 | 1 |
| 0 | 1 | 1 |
| 0 | 0 | 0 |
| 2 | 1 | 1 |
| 0 | 0 | 0 |
| 4 | 0 | 0 |
| 0 | 1 | 1 |
| 2 | 1 | 0 |
| 0 | 1 | 0 |
| 0 | 1 | 1 |
| 0 | 1 | 0 |
| 0 | 0 | 0 |
| 0 | 0 | 0 |
| 0 | 0 | 0 |
| 0 | 0 | 0 |
| 0 | 1 | 0 |
| 3 | 0 | 0 |
| 1 | 1 | 1 |
| 1 | 0 | 0 |
| 1 |  |  |
| 1 | 1 | 1 |
| 0 | 0 | 0 |
| 0 | 0 | 0 |
| 0 | 0 | 0 |
| 0 | 0 | 0 |
| 0 | 1 | 1 |
| 0 | 1 | 1 |
| 0 | 0 | 0 |
| 0 | 0 | 0 |
| 0 | 1 | 1 |
| 0 | 1 | 1 |
| 1 | 0 | 0 |
| 0 | 1 | 1 |
| 0 | 0 | 0 |
| 0 | 1 | 1 |
| 1 | 1 | 1 |
| 0 | 1 | 0 |
| 1 | 1 | 1 |
| 0 | 1 | 0 |
| 0 | 0 | 0 |
| 0 | 1 | 0 |
| 0 | 1 | 1 |
| 1 | 1 | 0 |
| 2 | 0 | 0 |
| 0 | 1 | 1 |
| 2 | 0 | 0 |
| 3 | 1 | 1 |
| 0 | 1 | 0 |
| 0 | 0 | 0 |
| 0 | 1 | 1 |
| 0 | 0 | 0 |
| 0 | 0 | 0 |
| 0 | 0 | 0 |
| 0 | 0 | 0 |
| 0 | 0 | 0 |
| 0 | 1 | 1 |
| 1 | 0 | 0 |
| 0 | 0 | 0 |
| 1 | 0 | 0 |
| 0 | 1 | 0 |
| 1 | 0 | 0 |
| 0 | 1 | 0 |
| 0 | 0 | 0 |
| 0 | 1 | 1 |
| 0 | 0 | 0 |
| 0 | 0 | 0 |
| 0 | 1 | 1 |
| 0 | 1 | 1 |
| 0 | 1 | 0 |
| 0 | 0 | 0 |
| 0 | 0 | 0 |
| 0 | 1 | 1 |
| 0 | 0 | 0 |
| 0 | 1 | 0 |
| 2 | 0 | 0 |
| 0 | 1 | 1 |
| 3 | 0 | 0 |
| 2 | 1 | 1 |
| 0 | 0 | 0 |
| 0 | 0 | 0 |
| 1 | 0 | 0 |
| 0 | 1 |  |
| 0 | 0 | 0 |
| 0 | 1 | 1 |
| 0 | 0 | 0 |
| 0 | 0 | 0 |
| 1 | 1 | 1 |
| 0 | 1 | 1 |
| 0 | 1 | 0 |
| 0 | 0 | 0 |
| 0 | 0 | 0 |
| 1 | 0 | 0 |
| 0 | 1 | 1 |
| 0 | 1 | 0 |
| 2 | 1 | 1 |
| 0 | 0 | 0 |
| 0 | 1 | 1 |
| 0 | 0 | 0 |
| 0 | 1 | 0 |
| 1 | 1 | 1 |
| 0 | 0 | 0 |
| 3 | 0 | 0 |
| 0 | 0 | 0 |
| 0 | 1 | 1 |
| 0 | 0 | 0 |
| 2 | 0 | 0 |
| 0 | 0 | 0 |
| 1 | 1 | 1 |
| 1 | 0 | 0 |
| 0 | 0 | 0 |
| 5 | 0 | 0 |
| 0 | 0 | 0 |
| 2 | 1 | 0 |
| 2 | 0 | 0 |
| 0 | 0 | 0 |
| 0 | 0 | 0 |
| 0 | 1 | 1 |
| 0 | 0 | 0 |
| 0 | 0 | 0 |
| 0 | 0 | 0 |
| 0 | 0 | 0 |
| 1 | 0 | 0 |
| 0 | 1 | 0 |
| 0 | 1 |  |
| 0 | 1 | 0 |
| 0 | 1 |  |
| 0 | 0 | 0 |
| 0 | 1 | 0 |
| 0 | 1 | 1 |
| 0 | 0 | 0 |
| 0 | 0 | 0 |
| 1 | 1 | 1 |
| 0 | 1 | 1 |
| 0 | 1 | 1 |
| 0 | 1 | 1 |
| 0 | 1 | 1 |
| 3 | 0 | 0 |
| 3 | 0 | 0 |
| 0 | 0 | 0 |
| 0 | 0 | 0 |
| 1 | 0 | 0 |
| 1 | 0 | 0 |
| 0 | 1 | 1 |
| 1 | 0 | 0 |
| 0 | 1 | 1 |
| 0 | 0 | 0 |
| 0 | 0 | 0 |
| 0 | 0 | 0 |
| 4 | 1 | 1 |
| 1 | 1 | 0 |
| 0 | 0 | 0 |
| 0 | 0 | 0 |
| 0 | 0 | 0 |
| 2 | 0 | 0 |
| 1 | 0 | 0 |
| 0 | 0 | 0 |
| 0 | 0 | 0 |
| 0 | 0 | 0 |
| 0 | 1 | 1 |
| 0 | 1 | 0 |
| 0 | 1 | 1 |
| 0 | 1 | 0 |
| 0 | 0 | 0 |
| 0 | 1 | 1 |
| 0 | 1 | 1 |
| 0 | 0 | 0 |
| 5 | 0 | 0 |
| 0 |  |  |
| 0 | 0 | 0 |
| 4 | 0 | 0 |
| 0 | 0 | 0 |
|  | 0 | 0 |
| 0 | 1 | 1 |
| 1 | 1 | 1 |
| 0 | 0 | 0 |
| 0 | 0 | 0 |
| 0 | 1 | 1 |
| 2 | 1 | 1 |
| 2 | 1 | 0 |
| 0 | 1 | 0 |
| 0 | 1 | 1 |
| 0 | 0 | 0 |
| 0 | 1 | 0 |
| 0 | 0 | 0 |
| 0 | 0 | 0 |
| 0 | 0 | 0 |
| 0 | 0 | 0 |
| 0 | 0 | 0 |
| 1 | 0 | 0 |
| 0 | 1 | 1 |
| 0 | 0 | 0 |
| 2 | 1 | 0 |
| 0 | 1 | 1 |
| 0 | 0 | 0 |
| 0 | 0 | 0 |
| 0 | 0 | 0 |
| 0 | 0 | 0 |
| 0 | 1 | 1 |
| 0 | 0 | 0 |
| 0 | 1 | 1 |
| 0 | 0 | 0 |
| 0 |  |  |
| 0 | 1 | 0 |
| 0 | 0 | 0 |
| 0 | 0 | 0 |
| 0 | 1 | 0 |
| 0 | 0 | 0 |
| 0 | 0 | 0 |
| 0 | 1 | 1 |
| 0 | 1 | 0 |
| 2 | 0 | 0 |
| 0 | 1 | 1 |
| 0 | 1 | 0 |
| 0 | 1 | 1 |
| 1 | 1 | 1 |
| 0 | 1 | 1 |
| 0 | 1 | 1 |
| 1 | 1 | 0 |
| 2 | 1 | 0 |
| 0 | 0 | 0 |
| 0 | 0 | 0 |
| 2 | 1 | 0 |
| 0 | 0 | 0 |
| 0 | 1 | 1 |
| 0 | 1 | 1 |
| 0 | 0 | 0 |
| 0 | 0 | 0 |
| 1 | 1 | 1 |
| 0 | 0 | 0 |
| 0 | 0 | 0 |
| 1 | 0 | 0 |
| 0 | 0 | 0 |
| 0 | 1 | 1 |
| 0 | 0 | 0 |
| 0 | 1 | 0 |
| 1 | 1 | 1 |
| 1 | 0 | 0 |
| 2 | 1 | 1 |
| 0 | 1 | 0 |
| 0 | 1 | 1 |
| 0 | 1 | 1 |
| 0 | 1 | 1 |
| 3 | 0 | 0 |
| 1 | 1 | 1 |
| 0 | 1 | 0 |
| 2 | 0 | 0 |
| 0 | 0 | 0 |
| 0 | 1 | 1 |
| 0 | 0 | 0 |
| 0 | 0 | 0 |
| 2 | 0 | 0 |
| 0 | 1 | 1 |
| 0 | 1 | 1 |
| 0 | 1 | 1 |
| 0 | 0 | 0 |
| 0 | 0 | 0 |
| 0 | 1 | 1 |
| 1 | 0 | 0 |
| 0 | 1 | 1 |
| 1 | 0 | 0 |
| 1 | 1 | 1 |
| 0 | 1 | 0 |
| 0 | 1 | 1 |
| 0 | 1 | 0 |
| 0 | 1 | 1 |
| 0 | 1 | 0 |
| 0 | 0 | 0 |
| 0 | 1 | 0 |
| 0 | 1 | 1 |
| 0 | 1 | 1 |
| 0 |  |  |
| 0 | 1 | 1 |
| 0 | 1 | 1 |
| 2 | 0 | 0 |
| 1 | 1 | 0 |
| 0 | 0 | 0 |
| 0 | 0 | 0 |
| 0 | 0 | 0 |
| 0 |  |  |
| 0 | 0 | 0 |
| 0 | 0 | 0 |
| 0 | 1 | 0 |
| 0 | 1 | 1 |
| 1 | 1 | 1 |
| 0 | 1 | 0 |
| 0 | 1 | 0 |
| 1 | 0 | 0 |
| 0 | 0 | 0 |
| 1 | 0 | 0 |
| 2 | 1 | 0 |
| 0 | 1 | 1 |
| 1 | 0 | 0 |
| 0 | 1 | 1 |
| 0 | 1 | 0 |
| 0 | 1 | 1 |
| 0 | 0 | 0 |
| 0 | 1 | 0 |
| 0 | 0 | 0 |
| 0 | 0 | 0 |
| 0 | 1 | 0 |
| 0 | 1 | 1 |
| 0 | 1 | 1 |
| 0 | 1 | 0 |
| 1 | 1 | 1 |
| 1 | 1 | 0 |
| 0 | 0 | 0 |
| 0 | 0 | 0 |
| 0 | 0 | 0 |
| 0 |  |  |
| 0 | 0 | 0 |
| 0 | 0 | 0 |
| 0 | 1 | 0 |
| 0 | 1 | 1 |
| 0 | 0 | 0 |
| 1 | 1 | 1 |
| 0 | 0 | 0 |
| 0 | 0 | 0 |
| 0 | 0 | 0 |
| 0 | 0 | 0 |
| 1 | 0 | 0 |
| 0 | 0 | 0 |
| 0 | 1 | 0 |
| 0 | 0 | 0 |
| 0 | 1 | 0 |
| 0 | 0 | 0 |
| 0 | 0 | 0 |
| 0 | 0 | 0 |
| 0 | 0 | 0 |
| 0 | 0 | 0 |
| 0 | 0 | 0 |
| 0 | 0 | 0 |
| 0 | 1 | 1 |
| 1 | 0 | 0 |
| 1 | 1 | 1 |
| 0 | 0 | 0 |
| 0 | 0 | 0 |
| 0 | 0 | 0 |
| 0 | 0 | 0 |
| 0 | 0 | 0 |
| 0 | 1 | 1 |
| 0 | 1 | 1 |
| 0 | 0 | 0 |
| 0 | 0 | 0 |
| 0 | 1 | 1 |
| 1 | 0 | 0 |
| 1 | 1 |  |
| 0 | 0 | 0 |
| 0 | 0 | 0 |
| 0 | 1 | 1 |
| 0 | 1 | 1 |
| 0 | 1 | 1 |
| 0 | 1 | 1 |
| 1 | 0 | 0 |
| 0 | 0 | 0 |
| 0 | 0 | 0 |
| 0 | 0 | 0 |
| 0 | 1 | 1 |
| 0 | 0 | 0 |
| 1 | 1 | 0 |
| 0 | 1 | 1 |
| 0 | 0 | 0 |
| 0 | 1 | 1 |
| 0 | 1 | 0 |
| 1 | 0 | 0 |
| 2 | 0 | 0 |
| 0 | 1 | 0 |
| 0 | 0 | 0 |
| 0 | 0 | 0 |
| 0 | 0 | 0 |
| 0 | 1 | 0 |
| 0 | 1 | 1 |
| 0 | 1 | 1 |
| 0 | 0 | 0 |
| 0 | 0 | 0 |
| 4 | 1 |  |
| 0 | 1 | 1 |
| 0 | 0 | 0 |
| 0 | 0 | 0 |
| 1 | 1 | 1 |
| 0 | 0 | 0 |
| 0 | 0 | 0 |
| 0 | 0 | 0 |
| 0 | 0 | 0 |
| 0 | 1 | 1 |
| 0 | 0 | 0 |
| 1 | 0 | 0 |
| 0 | 1 | 0 |
| 1 |  |  |
| 0 | 0 | 0 |
| 0 | 0 | 0 |
| 0 | 0 | 0 |
| 1 | 1 | 0 |
| 0 |  | 0 |
| 0 | 0 | 0 |
| 0 | 0 | 0 |
| 0 | 1 | 0 |
| 0 | 1 | 0 |
| 1 | 1 | 1 |
| 2 | 1 | 1 |
| 2 | 0 | 0 |
| 0 | 0 | 0 |
| 0 | 0 | 0 |
| 0 | 0 | 0 |
| 3 | 0 | 0 |
| 0 | 1 | 0 |
| 1 | 1 | 0 |
| 0 | 1 | 1 |
| 0 | 0 | 0 |
| 0 | 0 | 0 |
| 2 | 1 | 1 |
| 0 | 0 | 0 |
| 0 | 0 | 0 |
| 1 | 1 | 0 |
| 0 | 0 | 0 |
| 0 | 0 | 0 |
| 1 | 1 | 0 |
| 0 | 0 | 0 |
| 0 | 1 | 1 |
| 1 | 1 | 1 |
| 0 | 0 | 0 |
| 0 | 0 | 0 |
| 0 | 1 | 1 |
| 0 |  |  |
| 0 | 0 | 0 |
| 1 | 1 | 1 |
| 1 | 0 | 0 |
| 0 | 0 | 0 |
| 1 | 0 | 0 |
| 1 | 1 | 1 |
| 0 | 1 | 0 |
| 0 | 0 | 0 |
| 0 | 0 | 0 |
| 0 | 1 | 1 |
| 0 | 1 | 1 |
| 1 | 0 | 0 |
| 0 | 0 | 0 |
| 0 | 1 | 1 |
| 0 | 0 | 0 |
| 0 | 0 | 0 |
| 0 | 1 | 1 |
| 0 | 0 | 0 |
| 0 | 0 | 0 |
| 0 | 1 | 0 |
| 1 | 1 | 1 |
| 0 | 0 | 0 |
| 3 | 1 | 1 |
| 0 | 1 | 1 |
| 0 | 0 | 0 |
| 0 | 1 | 1 |
| 0 | 0 | 0 |
| 0 | 1 | 1 |
| 1 | 1 | 1 |
| 0 | 0 | 0 |
| 1 | 0 | 0 |
| 0 | 1 | 1 |
| 1 | 1 | 1 |
| 0 | 1 | 1 |
| 2 | 1 | 0 |
| 0 | 0 | 0 |
| 1 |  | 0 |
| 0 | 1 | 1 |
| 0 | 0 | 0 |
| 0 | 0 | 0 |
| 0 | 0 | 0 |
| 0 | 0 | 0 |
| 1 | 1 | 1 |
| 0 | 1 | 0 |
| 0 | 0 | 0 |
| 0 | 0 | 0 |
| 0 | 0 | 0 |
| 0 | 0 | 0 |
| 0 | 0 | 0 |
| 0 | 1 | 0 |
| 0 | 1 | 1 |
| 0 | 1 | 0 |
| 0 | 1 | 1 |
| 0 | 0 | 0 |
| 0 | 0 | 0 |
| 0 | 1 | 0 |
| 1 | 0 | 0 |
| 0 | 0 | 0 |
| 0 | 0 | 0 |
| 0 | 1 | 1 |
| 2 | 0 | 0 |
| 0 | 0 | 0 |
| 0 | 0 | 0 |
| 0 | 0 | 0 |
| 0 | 0 | 0 |
| 0 | 1 | 0 |
| 0 | 1 | 0 |
| 0 | 1 | 1 |
| 0 | 0 | 0 |
| 0 | 1 | 0 |
| 0 | 0 | 0 |
| 0 | 0 | 0 |
| 0 | 0 | 0 |
| 0 | 1 | 1 |
| 2 | 1 | 1 |
| 2 | 0 | 0 |
| 0 | 1 | 1 |
| 0 | 0 | 0 |
| 0 | 1 | 0 |
| 0 | 1 | 1 |
| 2 | 0 | 0 |
| 0 | 1 | 1 |
| 0 | 0 | 0 |
| 0 | 1 | 1 |
| 0 | 0 | 0 |
| 1 | 1 | 0 |
| 0 | 0 | 0 |
| 1 | 0 | 0 |
| 1 | 0 | 0 |
| 0 | 0 | 0 |
| 0 |  |  |
| 0 | 1 | 1 |
| 0 | 1 | 1 |
| 0 | 0 | 0 |
| 0 | 1 | 1 |
| 2 | 1 | 1 |
| 1 | 1 | 1 |
| 0 | 1 | 1 |
| 2 | 0 | 0 |
| 0 | 1 | 1 |
| 0 | 1 | 1 |
| 0 | 0 | 0 |
| 0 | 0 | 0 |
| 1 | 0 | 0 |
| 0 | 0 | 0 |
| 0 | 1 | 1 |
| 2 | 0 | 0 |
| 0 | 1 | 0 |
| 0 | 1 | 0 |
| 0 | 0 | 0 |
| 0 | 1 | 1 |
| 0 | 1 | 1 |
| 0 | 1 | 1 |
| 0 | 1 | 1 |
| 0 | 1 | 1 |
| 1 | 1 | 1 |
| 0 | 0 | 0 |
| 0 | 1 | 1 |
| 1 | 1 | 1 |
| 0 | 0 | 0 |
| 0 | 1 | 1 |
| 0 | 1 | 1 |
| 2 | 0 | 0 |
| 2 | 0 | 0 |
| 1 | 1 | 1 |
| 0 | 0 | 0 |
| 0 | 1 | 0 |
| 1 | 0 | 0 |
| 0 | 1 | 0 |
| 0 | 1 | 1 |
| 0 | 0 | 0 |
| 0 |  |  |
| 1 | 0 | 0 |
| 0 | 1 | 1 |
| 0 | 1 | 1 |
| 1 | 0 | 0 |
| 0 | 0 | 0 |
| 0 | 0 | 0 |
| 0 | 0 | 0 |
| 2 | 0 | 0 |
| 2 | 0 | 0 |
| 0 | 0 | 0 |
| 0 | 0 | 0 |
| 1 | 0 | 0 |
| 0 | 0 | 0 |
| 1 | 0 | 0 |
| 0 | 1 | 1 |
| 0 | 1 | 1 |
| 1 | 0 | 0 |
| 0 | 0 | 0 |
| 0 | 0 | 0 |
| 1 | 1 | 0 |
| 0 | 1 | 0 |
| 1 | 0 | 0 |
| 0 | 0 | 0 |
| 0 | 0 | 0 |
| 0 | 0 | 0 |
| 0 | 1 | 1 |
| 0 | 0 | 0 |
| 0 | 0 | 0 |
| 0 | 1 | 1 |
| 0 | 0 | 0 |
| 0 | 1 | 0 |
| 1 | 0 | 0 |
| 0 | 0 | 0 |
| 0 | 0 | 0 |
| 0 | 0 | 0 |
| 0 | 1 | 0 |
| 0 | 1 | 1 |
| 2 | 1 | 0 |
| 0 | 0 | 0 |
| 0 | 1 | 0 |
| 0 | 1 | 0 |
| 0 | 0 | 0 |
| 0 | 0 | 0 |
| 0 | 1 | 1 |
| 1 | 1 | 1 |
| 0 | 1 | 1 |
| 0 | 1 | 1 |
| 0 | 1 | 1 |
| 0 | 0 | 0 |
| 0 | 1 | 1 |
| 1 | 0 | 0 |
| 0 | 1 | 0 |
| 0 | 0 | 0 |
| 0 | 1 | 0 |
| 0 | 0 | 0 |
| 0 |  |  |
| 0 | 0 | 0 |
| 0 | 0 | 0 |
| 1 | 0 | 0 |
| 0 | 1 | 1 |
| 0 | 1 | 1 |
| 0 | 0 | 0 |
| 0 | 1 | 1 |
| 0 | 0 | 0 |
| 0 | 0 | 0 |
| 2 | 0 | 0 |
| 0 | 1 | 0 |
| 0 | 1 | 1 |
| 0 | 0 | 0 |
| 0 | 1 | 1 |
| 0 | 1 | 1 |
| 3 | 1 | 1 |
| 0 | 0 | 0 |
| 1 | 1 | 1 |
| 1 | 1 | 1 |
| 0 | 0 | 0 |
| 0 | 1 | 1 |
| 1 | 0 | 0 |
| 0 | 0 | 0 |
| 0 | 0 | 0 |
| 0 | 0 | 0 |
| 0 | 1 | 1 |
| 1 | 1 | 0 |
| 0 | 0 | 0 |
| 0 | 1 | 0 |
| 0 | 1 | 1 |
| 2 | 0 | 0 |
| 0 | 1 | 1 |
| 0 | 1 | 1 |
| 1 | 0 | 0 |
| 0 |  |  |
| 0 | 1 | 1 |
| 1 | 1 | 1 |
| 1 | 1 | 0 |
| 0 | 0 | 0 |
| 0 | 1 | 1 |
| 0 | 1 | 0 |
| 0 | 1 | 1 |
| 1 | 1 | 0 |
| 2 | 1 | 1 |
| 0 | 1 | 1 |
| 0 | 1 | 0 |
| 0 | 0 | 0 |
| 0 | 1 | 1 |
| 0 | 0 | 0 |
| 0 | 1 | 1 |
| 0 | 1 | 1 |
| 2 | 1 | 1 |
| 0 | 0 | 0 |
| 0 | 0 | 0 |
| 1 | 1 | 0 |
| 0 | 0 | 0 |
| 1 | 0 | 0 |
| 0 | 1 | 1 |
| 0 | 1 | 1 |
| 2 | 1 | 1 |
| 0 | 1 | 0 |
| 0 | 0 | 0 |
| 0 | 0 | 0 |
| 0 | 1 | 0 |
| 0 | 0 | 0 |
| 0 | 0 | 0 |
| 0 | 1 | 0 |
| 0 | 0 | 0 |
| 0 | 1 | 1 |
| 0 | 1 | 1 |
| 2 | 1 | 1 |
| 0 | 1 | 1 |
| 0 | 1 | 0 |
| 0 | 0 | 0 |
| 0 | 1 | 1 |
| 0 | 1 | 1 |
| 3 | 0 | 0 |
| 1 | 0 | 0 |
| 3 | 0 | 0 |
| 0 | 0 | 0 |
| 0 | 1 | 1 |
| 1 | 1 | 1 |
| 0 | 1 | 0 |
| 0 | 1 | 1 |
| 0 | 1 | 0 |
| 0 | 0 | 0 |
| 0 | 1 | 1 |
| 0 | 0 | 0 |
| 0 | 1 | 0 |
| 1 | 0 | 0 |
| 0 | 1 | 0 |
| 0 | 0 | 0 |
| 1 | 0 | 0 |
| 0 | 1 | 1 |
| 0 | 0 | 0 |
| 0 | 1 | 0 |
| 1 | 0 | 0 |
| 0 | 0 | 0 |
| 0 | 1 | 1 |
| 0 | 1 | 0 |
| 4 | 0 | 0 |
| 0 | 0 | 0 |
| 0 | 0 | 0 |
| 0 | 0 | 0 |
| 0 | 0 | 0 |
| 1 | 0 | 0 |
| 2 | 1 | 1 |
| 0 | 1 | 1 |
| 0 | 1 | 0 |
| 0 | 0 | 0 |
| 0 | 1 | 0 |
| 1 | 0 | 0 |
| 0 | 0 | 0 |
| 0 | 0 | 0 |
| 0 | 1 | 1 |
| 2 | 1 | 0 |
| 0 | 0 | 0 |
| 0 | 1 | 1 |
| 0 | 1 | 0 |
| 0 | 0 | 0 |
| 0 | 1 | 0 |
| 0 | 0 | 0 |
| 0 | 0 | 0 |
| 1 | 1 | 1 |
| 0 | 0 | 0 |
| 0 | 1 | 1 |
| 1 | 0 | 0 |
| 0 | 1 | 1 |
| 0 | 1 | 1 |
| 0 | 0 | 0 |
| 0 | 1 | 1 |
| 0 | 1 | 0 |
| 0 | 0 | 0 |
| 1 | 1 | 1 |
| 0 | 0 | 0 |
| 0 | 1 | 1 |
| 0 | 1 | 1 |
| 1 | 1 | 1 |
| 0 | 0 | 0 |
| 0 | 0 | 0 |
| 0 | 1 | 1 |
| 0 | 0 | 0 |
| 0 | 1 | 1 |
| 1 | 0 | 0 |
| 0 | 0 | 0 |
| 0 | 0 | 0 |
| 0 | 1 | 1 |
| 0 | 0 | 0 |
| 1 | 1 | 0 |
| 0 | 0 | 0 |
| 0 | 1 | 0 |
| 0 | 0 | 0 |
| 0 | 1 | 1 |
| 1 | 0 | 0 |
| 0 | 1 | 0 |
| 0 | 1 | 1 |
| 0 | 1 | 0 |
| 0 | 0 | 0 |
| 0 | 0 | 0 |
| 1 | 0 | 0 |
| 0 | 1 | 1 |
| 0 | 1 | 1 |
| 0 | 0 | 0 |
| 0 | 1 | 0 |
| 0 | 0 | 0 |
| 0 | 0 | 0 |
| 0 | 1 | 1 |
| 0 | 1 | 0 |
| 0 | 0 | 0 |
| 1 | 0 | 0 |
| 0 | 1 | 1 |
| 0 | 0 | 0 |
| 0 | 1 | 1 |
| 0 | 1 | 1 |
| 2 | 0 | 0 |
| 0 | 0 | 0 |
|  | 1 | 1 |
| 0 | 1 | 0 |
| 0 | 1 | 0 |
| 0 | 0 | 0 |
| 0 | 0 | 0 |
| 0 | 1 | 0 |
| 0 | 0 | 0 |
| 0 | 1 | 0 |
| 0 | 0 | 0 |
| 0 | 0 | 0 |
| 0 | 1 | 1 |
| 0 | 0 | 0 |
| 1 | 1 | 0 |
| 0 | 0 | 0 |
| 0 | 0 | 0 |
| 0 | 0 | 0 |
| 0 | 1 | 1 |
| 1 | 0 | 0 |
| 0 | 1 | 0 |
| 0 | 0 | 0 |
| 0 | 0 | 0 |
| 2 | 0 | 0 |
| 0 | 0 | 0 |
| 0 | 1 | 1 |
| 3 | 0 | 0 |
| 3 | 0 | 0 |
| 0 | 0 | 0 |
| 2 | 0 | 0 |
| 1 | 0 | 0 |
| 0 | 0 | 0 |
| 0 | 1 | 0 |
| 1 | 0 | 0 |
| 0 | 1 | 0 |
| 0 | 1 | 0 |
| 0 | 1 | 0 |
| 1 | 1 | 1 |
| 0 | 1 | 1 |
| 1 | 0 | 0 |
| 0 | 1 | 1 |
| 0 | 0 | 0 |
| 2 | 0 | 0 |
| 2 | 0 | 0 |
| 0 | 1 | 0 |
