# Supplementary material for: Pet-keeping in early life reduces the risk of allergy in a dose-dependent fashion
Source: PLoS One. 2018 Dec 19;13(12):e0208472. doi: 10.1371/journal.pone.0208472 (PMC6300190; doi:10.1371/journal.pone.0208472)
Supplement: S5 Table — (DOCX) [file pone.0208472.s005.docx]

| Allergy last yr | Allergy ever | Pets | BHR | FEV1/FVC | Mother allergy | Father allergy | B-Eos | Eosproc | Total IgE | SPT birch | SPT grass | SPT cat | SPT dog | SPT horse | SPT |
| --- | --- | --- | --- | --- | --- | --- | --- | --- | --- | --- | --- | --- | --- | --- | --- |
| 1 | 1 | 0 | 2,81 | 90,00 | 1 | 1 | 0,10 | 1,10 | 22,00 | 0 | 0 | 0 | 0 | 0 | 0 |
|  |  | 1 |  |  | 0 | 1 |  |  |  |  |  |  |  |  |  |
| 1 | 1 | 0 | 27,30 | 80,00 | 1 | 0 | 0,10 | 2,08 | 5,00 | 0 | 0 | 0 | 0 | 0 | 0 |
| 0 | 1 | 0 | 24,30 | 85,00 | 0 | 1 | 0,30 | 4,92 | 15,00 | 0 | 0 | 0 | 0 | 0 | 0 |
| 0 | 0 | 0 | 49,70 | 92,00 | 1 | 0 | 0,10 | 1,47 | 2,00 | 0 | 0 | 0 | 0 | 0 | 0 |
| 1 | 1 | 0 | 21,77 | 86,00 | 0 | 1 | 0,09 | 0,81 | 40,00 | 0 | 0 | 0 | 0 | 0 | 0 |
|  |  | 0 |  |  | 0 | 0 |  |  |  |  |  |  |  |  |  |
|  |  | 0 |  |  | 0 | 0 |  |  |  |  |  |  |  |  |  |
| 0 | 0 | 0 | 4,20 | 88,00 | 0 | 0 | 0,30 | 4,55 | 35,00 | 0 | 0 | 0 | 0 | 0 | 0 |
|  |  | 0 |  |  | 0 | 0 |  |  |  |  |  |  |  |  |  |
|  |  | 0 |  |  | 0 | 0 |  |  |  |  |  |  |  |  |  |
| 0 | 1 | 0 | 88,50 | 84,00 | 0 | 0 | 0,70 | 10,61 | 65,00 | 0 | 0 | 0 | 0 | 0 | 0 |
| 0 | 0 | 0 | 10,10 | 91,00 | 1 | 1 | 0,10 | 1,33 | 5,00 | 0 | 0 | 0 | 0 | 0 | 0 |
| 0 | 0 | 0 | 7,15 | 95,00 | 1 | 0 |  |  | 19,00 | 0 | 0 | 0 | 0 | 0 | 0 |
| 0 | 0 | 0 | 67,90 | 91,00 |  |  | 0,30 | 5,56 | 40,00 | 0 | 0 | 0 | 0 | 0 | 0 |
| 0 | 0 | 0 | 22,70 | 85,00 | 0 | 0 | 0,20 | 4,00 | 7,00 | 0 | 0 | 0 | 0 | 0 | 0 |
|  |  | 0 |  |  | 0 | 1 |  |  |  |  |  |  |  |  |  |
| 1 | 1 | 0 | 22,80 | 95,00 | 0 | 1 | 0,10 | 1,10 | 50,00 | 0 | 0 | 0 | 0 | 0 | 0 |
| 1 | 1 | 0 | 77,50 | 87,00 | 1 | 0 | 0,60 | 8,82 | 85,00 | 0 | 0 | 0 | 0 | 0 | 0 |
| 0 | 1 | 0 | 11,80 | 79,00 | 0 | 1 | 0,50 | 6,33 | 65,00 | 0 | 0 | 0 | 0 | 0 | 0 |
|  |  | 0 |  |  | 0 | 1 |  |  |  |  |  |  |  |  |  |
| 0 | 0 | 0 | 26,20 | 89,00 | 0 | 1 | 0,10 | 1,56 | 60,00 | 0 | 0 | 0 | 0 | 0 | 0 |
| 0 | 1 | 1 | 136,00 | 76,00 | 0 | 1 | 0,20 | 3,17 | 140,00 | 0 | 0 | 0 | 0 | 0 | 0 |
| 1 | 1 | 0 | 206,00 | 94,00 | 1 | 0 | 0,70 | 10,14 | 220,00 | 4 | 5 | 8 | 6 | 9 | 1 |
| 0 | 0 | 0 | 58,80 | 92,00 | 1 | 1 | 0,30 | 4,62 | 200,00 | 0 | 0 | 0 | 0 | 0 | 0 |
|  |  | 0 |  |  | 1 | 0 |  |  |  |  |  |  |  |  |  |
|  |  | 0 |  |  | 1 | 0 |  |  |  |  |  |  |  |  |  |
| 0 | 0 | 1 | 1,19 | 90,00 | 1 | 0 | 0,30 | 3,80 | 35,00 | 0 | 0 | 0 | 0 | 0 | 0 |
| 1 | 1 | 1 |  | 83,00 | 1 | 0 | 0,20 | 3,13 | 18,00 | 0 | 0 | 0 | 0 | 0 | 0 |
|  |  | 0 |  |  | 0 | 1 |  |  |  |  |  |  |  |  |  |
| 0 | 0 | 1 | 5,46 | 84,00 | 1 | 0 | 0,70 | 11,48 | 140,00 | 0 | 0 | 0 | 0 | 0 | 0 |
|  |  | 0 |  |  | 1 | 1 |  |  |  |  |  |  |  |  |  |
| 1 | 1 | 0 |  | 47,00 | 0 | 1 | 0,50 | 9,62 | 1200,00 | 6 | 5 | 0 | 0 | 6 | 1 |
| 0 | 0 | 0 |  | 79,00 | 0 | 1 | 0,30 | 5,00 | 55,00 | 0 | 0 | 0 | 0 | 0 | 0 |
| 0 | 0 | 0 | 77,70 | 83,00 | 0 | 0 | 0,70 | 8,97 | 80,00 | 0 | 0 | 0 | 0 | 0 | 0 |
| 0 | 0 | 1 | 43,20 | 83,00 | 1 | 1 | 0,20 | 2,90 | 48,00 | 0 | 2 | 0 | 0 | 0 | 0 |
| 0 | 0 | 0 |  |  | 1 | 1 |  |  |  |  |  |  |  |  |  |
| 1 | 1 | 0 | 2,84 | 89,00 | 0 | 0 | 0,08 | 1,29 | 26,00 | 0 | 0 | 0 | 0 | 0 | 0 |
| 0 | 0 | 0 |  |  | 1 | 0 |  |  |  |  |  |  |  |  |  |
| 1 | 1 | 0 | 144,00 | 75,00 | 1 | 0 | 0,20 | 2,67 | 380,00 | 0 | 3 | 6 | 4 | 1 | 1 |
| 1 | 1 | 1 | 61,20 | 88,00 | 0 | 0 | 0,60 | 9,68 | 580,00 | 0 | 0 | 0 | 0 | 0 | 0 |
| 0 | 0 | 0 | 57,10 | 95,00 | 1 | 0 | 0,06 | 0,60 | 18,00 | 0 | 0 | 0 | 0 | 0 | 0 |
| 1 | 1 | 0 | 0,95 | 92,00 | 1 | 0 | 0,20 | 3,77 | 200,00 | 4 | 0 | 7 | 0 | 5 | 1 |
| 0 | 0 | 0 | 1,62 | 84,00 | 1 | 0 | 0,40 | 7,69 | 50,00 | 0 | 0 | 0 | 4 | 0 | 1 |
|  |  | 0 |  |  | 1 | 0 |  |  |  |  |  |  |  |  |  |
| 1 | 1 | 0 | 46,80 | 86,00 | 0 | 1 | 0,10 | 1,89 | 20,00 | 0 | 0 | 0 | 0 | 0 | 0 |
| 0 | 0 | 0 | 7,10 | 87,00 | 0 | 1 | 0,10 | 1,22 | 2,60 | 0 | 0 | 0 | 0 | 0 | 0 |
| 0 | 0 | 0 | 27,00 | 86,00 | 0 | 1 | 0,80 | 12,90 | 35,00 | 0 | 0 | 0 | 0 | 0 | 1 |
| 0 | 0 | 2 |  |  | 1 | 0 |  |  |  |  |  |  |  |  |  |
| 0 | 0 | 0 | 9,25 | 77,00 | 0 | 1 | 0,30 | 4,62 | 11,00 | 0 | 0 | 0 | 0 | 0 | 0 |
| 1 | 1 | 0 | 45,70 |  | 1 | 0 | 0,30 | 5,45 | 330,00 | 5 | 7 | 8 | 2 | 5 | 1 |
| 1 | 1 | 1 | 23,50 | 86,00 | 1 | 0 | 0,30 | 5,17 | 170,00 | 7 | 0 | 0 | 0 | 0 | 1 |
| 0 | 0 | 1 | 25,60 | 89,00 | 0 | 1 | 0,20 | 2,56 | 300,00 | 0 | 0 | 0 | 0 | 0 | 0 |
| 1 | 1 | 0 | 1,08 | 95,00 | 0 | 1 | 0,30 | 3,23 | 150,00 | 8 | 0 | 0 | 4 | 0 | 1 |
| 1 | 1 | 0 | 131,00 | 73,00 | 1 | 0 | 1,00 | 13,33 | 65,00 | 4 | 9 | 4 | 3 | 0 | 1 |
| 0 | 0 | 0 |  |  | 1 | 1 |  |  |  |  |  |  |  |  |  |
| 0 | 0 | 0 | 6,72 | 89,00 | 1 | 0 | 0,10 | 1,67 | 20,00 | 0 | 0 | 0 | 0 | 0 | 0 |
| 1 | 1 | 0 |  |  | 0 | 1 | 0,20 | 2,53 | 11,00 | 0 | 0 | 0 | 0 | 0 | 0 |
| 0 | 0 | 0 | 246,00 | 78,00 | 0 | 1 | 0,10 | 1,89 | 2,10 | 0 | 0 | 0 | 0 | 0 | 0 |
| 0 | 0 | 0 | 9,17 | 86,00 | 1 | 1 | 0,08 | 1,63 | 37,00 | 0 | 0 | 0 | 0 | 0 | 0 |
| 1 | 1 | 0 | 122,00 | 89,00 | 1 | 0 | 0,70 | 10,61 | 380,00 | 0 | 0 | 0 | 0 | 0 | 0 |
| 0 | 0 | 0 | 671,00 | 66,00 | 1 | 0 | 0,30 | 4,62 | 5,90 | 0 | 0 | 0 | 0 | 0 | 0 |
| 0 | 0 | 0 | 2,88 | 85,00 | 1 | 0 | 0,20 | 4,44 | 17,00 | 0 | 0 | 0 | 0 | 0 | 0 |
|  |  | 0 |  |  | 0 | 1 |  |  |  |  |  |  |  |  |  |
| 0 | 0 | 0 | 273,00 | 86,00 | 1 | 0 | 0,10 | 2,13 | 50,00 | 0 | 0 | 0 | 0 | 0 | 1 |
| 0 | 0 | 1 | 45,97 | 88,00 | 1 | 0 | 0,20 | 3,03 | 91,00 | 0 | 0 | 0 | 0 | 0 | 0 |
| 0 | 0 | 0 |  |  | 0 | 1 |  |  |  |  |  |  |  |  |  |
| 0 | 0 | 2 | 4,19 | 94,00 | 1 | 0 | 0,10 | 1,59 | 57,00 | 0 | 0 | 0 | 0 | 0 | 0 |
| 0 | 0 | 0 | 2,33 | 84,00 | 1 | 0 | 0,10 | 0,90 | 11,00 | 0 | 0 | 0 | 0 | 0 | 0 |
| 1 | 1 | 2 | 28,83 | 78,00 | 1 | 0 | 0,30 | 2,40 | 210,00 | 5 | 0 | 0 | 0 | 0 | 1 |
| 1 | 1 | 0 | 639,00 | 70,00 | 1 | 0 | 0,60 | 6,98 | 1100,00 | 5 | 3 | 0 | 0 | 0 | 1 |
| 1 | 1 | 1 | 1,48 | 85,00 | 1 | 0 | 0,40 | 6,06 | 35,00 | 0 | 3 | 0 | 0 | 0 | 1 |
|  |  | 0 |  |  | 1 | 0 |  |  |  |  |  |  |  |  |  |
| 0 | 1 | 0 | 31,79 | 75,00 | 1 | 0 | 0,30 | 5,88 | 150,00 | 0 | 0 | 0 | 0 | 0 | 1 |
| 0 | 0 | 0 | 120,00 | 85,00 | 0 | 1 | 0,10 | 1,32 | 8,00 | 0 | 0 | 0 | 0 | 0 | 0 |
| 1 | 1 | 0 | 1,60 | 91,00 | 1 | 1 | 0,20 | 4,00 | 470,00 | 0 | 5 | 0 | 0 | 0 | 1 |
|  |  | 0 |  |  | 1 | 0 |  |  |  |  |  |  |  |  |  |
| 0 | 0 | 0 | 12,08 | 89,00 | 1 | 0 | 0,10 | 1,27 | 6,00 | 0 | 0 | 0 | 0 | 0 | 0 |
| 0 | 0 | 0 | 21,33 | 87,00 | 1 | 1 | 0,40 | 5,33 | 110,00 | 3 | 3 | 0 | 0 | 5 | 1 |
| 0 | 0 | 0 | 7,67 | 92,00 | 1 | 0 | 0,07 | 1,03 | 4,00 | 0 | 0 | 0 | 0 | 0 | 0 |
| 1 | 1 | 0 | 20,89 | 84,00 | 1 | 0 | 1,00 | 9,71 | 920,00 | 7 | 8 | 9 | 4 | 0 | 1 |
| 1 | 1 | 0 |  |  | 1 | 0 | 0,90 | 14,52 | 475,00 | 3 | 3 | 4 | 1 | 4 | 1 |
| 1 | 1 | 0 | 129,85 | 62,00 | 1 | 1 | 0,20 | 2,70 | 28,00 | 0 | 0 | 0 | 0 | 0 | 0 |
| 1 | 1 | 0 | 14,40 | 89,00 | 0 | 1 | 0,10 | 1,30 | 31,00 | 3 | 0 | 8 | 1 | 7 | 1 |
| 1 | 1 | 0 | 81,80 |  | 1 | 0 | 0,30 | 5,45 | 94,00 | 0 | 0 | 0 | 0 | 0 | 1 |
| 1 | 1 | 0 | 12,65 | 89,00 | 0 | 1 | 0,20 | 3,45 | 61,00 | 0 | 0 | 0 | 0 | 0 | 0 |
|  |  | 1 |  |  | 0 | 1 |  |  |  |  |  |  |  |  |  |
| 1 | 1 | 0 | 25,67 | 98,00 | 1 | 0 | 0,07 | 1,25 | 88,00 | 0 | 0 | 0 | 0 | 0 | 0 |
| 1 | 1 | 0 | 61,64 | 78,00 | 1 | 0 | 0,20 | 2,33 | 29,00 | 6 | 3 | 3 | 0 | 0 | 1 |
| 1 | 1 | 0 | 12,69 | 83,00 | 1 | 1 | 0,30 | 3,66 | 120,00 | 4 | 2 | 0 | 0 | 0 | 1 |
| 1 | 1 | 0 | 46,85 | 83,00 | 0 | 1 | 0,20 | 4,17 | 420,00 | 6 | 3 | 5 | 0 | 0 | 1 |
|  |  | 1 |  |  | 1 | 0 |  |  |  |  |  |  |  |  |  |
|  |  | 0 |  |  | 1 | 0 |  |  |  |  |  |  |  |  |  |
| 0 | 0 | 0 | 46,84 | 87,00 | 0 | 1 | 0,10 | 1,75 | 16,00 | 0 | 0 | 0 | 0 | 0 | 0 |
| 0 | 0 | 0 | 20,22 |  | 1 | 0 | 0,06 | 1,09 | 170,00 | 0 | 0 | 0 | 0 | 0 | 0 |
| 1 | 1 | 0 | 167,90 | 90,00 | 1 | 0 | 0,04 | 0,63 | 150,00 | 0 | 6 | 9 | 0 | 0 | 1 |
| 0 | 0 | 0 |  |  | 1 | 1 |  |  |  |  |  |  |  |  |  |
| 0 | 0 | 0 | 155,40 | 65,00 | 0 | 1 | 0,30 | 5,56 | 49,00 | 0 | 0 | 0 | 0 | 0 | 0 |
| 0 | 0 | 0 | 3,12 | 88,00 | 0 | 1 | 0,40 | 6,06 | 59,00 | 0 | 0 | 0 | 0 | 0 | 0 |
| 1 | 1 | 0 | 63,01 | 88,00 | 0 | 1 | 0,60 | 7,69 | 110,00 | 4 | 0 | 0 | 0 | 0 | 1 |
|  |  | 0 |  |  | 1 | 1 |  |  |  |  |  |  |  |  |  |
| 0 | 0 | 0 |  |  | 0 | 0 |  |  |  |  |  |  |  |  |  |
|  |  | 0 |  |  | 0 | 0 |  |  |  |  |  |  |  |  |  |
|  |  | 0 |  |  | 0 | 1 |  |  |  |  |  |  |  |  |  |
| 0 | 0 | 0 | 2,90 | 91,00 | 1 | 0 | 0,20 | 3,45 | 41,00 | 0 | 0 | 4 | 0 | 0 | 1 |
| 0 | 0 | 0 | 60,41 | 92,00 | 1 | 1 | 0,03 | 0,48 | 4,00 | 0 | 0 | 0 | 0 | 0 | 0 |
| 1 | 1 | 2 | 23,36 | 84,00 | 0 | 1 | 0,30 | 4,00 | 130,00 | 6 | 0 | 0 | 0 | 0 | 1 |
| 0 | 0 | 0 |  |  | 0 | 1 |  |  |  |  |  |  |  |  |  |
| 0 | 0 | 0 | 27,94 | 78,00 | 1 | 0 | 1,70 | 17,17 | 280,00 | 0 | 0 | 0 | 0 | 0 | 0 |
| 0 | 0 | 0 | 63,30 | 95,00 | 1 | 0 | 0,50 | 6,94 | 170,00 | 0 | 0 | 0 | 0 | 0 | 0 |
| 1 | 1 | 0 | 13,64 | 84,00 | 0 | 0 | 0,20 | 3,13 |  | 0 | 5 | 6 | 4 | 0 | 1 |
| 0 | 0 | 0 | 0,63 | 82,00 | 1 | 0 | 0,20 | 2,94 | 20,00 | 0 | 0 | 0 | 0 | 0 | 0 |
| 1 | 1 | 0 | 282,60 | 79,00 | 1 | 0 | 0,50 | 8,20 | 34,00 | 0 | 0 | 0 | 0 | 0 | 0 |
| 1 | 1 | 0 | 132,50 | 66,00 | 0 | 1 | 0,10 | 2,56 | 31,00 | 0 | 0 | 0 | 0 | 0 | 0 |
| 1 | 1 | 0 | 2,69 | 79,00 | 0 | 1 | 0,30 | 5,00 | 120,00 | 3 | 2 | 4 | 2 | 0 | 1 |
| 1 | 1 | 0 | 27,11 | 77,00 | 0 | 1 | 0,30 | 4,62 | 11,00 | 0 | 0 | 0 | 0 | 0 | 0 |
| 0 | 1 | 0 |  |  | 0 | 1 |  |  |  |  |  |  |  |  |  |
| 1 | 1 | 0 |  |  | 1 | 0 |  |  |  |  |  |  |  |  |  |
| 0 | 0 | 0 |  |  | 1 | 0 |  |  |  |  |  |  |  |  |  |
| 1 | 1 | 0 | 13,80 | 89,00 | 1 | 0 | 0,20 | 2,63 | 6,30 | 0 | 0 | 0 | 0 | 0 | 0 |
| 0 | 0 | 0 | 186,04 | 85,00 | 0 | 0 | 0,60 | 10,71 | 47,00 | 0 | 0 | 0 | 0 | 0 | 0 |
| 0 | 0 | 0 |  |  | 0 | 0 |  |  |  |  |  |  |  |  |  |
| 0 | 0 | 0 |  |  | 0 | 1 |  |  |  |  |  |  |  |  |  |
| 0 | 0 | 1 |  |  | 1 | 0 |  |  |  |  |  |  |  |  |  |
| 0 | 0 | 0 | 16,70 | 91,00 | 0 | 0 | 0,12 | 2,45 | 8,70 | 0 | 0 | 0 | 0 | 0 | 0 |
| 1 | 1 | 0 | 23,55 | 83,00 | 0 | 1 | 0,10 | 1,43 | 140,00 | 4 | 5 | 0 | 0 | 0 | 1 |
| 0 | 0 | 0 | 48,48 | 75,00 | 0 | 1 | 0,09 | 1,61 | 74,00 | 0 | 0 | 0 | 0 | 0 | 0 |
| 0 | 0 | 0 |  |  | 1 | 0 |  |  |  |  |  |  |  |  |  |
| 0 | 0 | 0 | 101,44 | 91,00 | 0 | 1 | 0,10 | 1,27 | 20,00 | 0 | 0 | 0 | 0 | 0 | 0 |
| 0 | 0 | 0 | 79,94 | 76,00 | 0 | 1 | 0,50 | 8,33 | 230,00 | 0 | 4 | 0 | 2 | 0 | 1 |
| 1 | 1 | 0 | 12,96 | 77,00 | 1 | 0 | 0,20 | 5,41 | 72,00 | 0 | 0 | 0 | 0 | 0 | 0 |
| 1 | 1 | 0 | 789,30 | 78,00 | 1 | 0 | 0,80 | 12,31 | 500,00 | 0 | 0 | 0 | 0 | 0 | 0 |
| 0 | 0 | 0 | 21,58 | 90,00 | 0 | 1 | 0,30 | 5,26 |  | 0 | 0 | 0 | 0 | 0 | 0 |
| 0 | 1 | 0 |  |  | 0 | 1 |  |  |  |  |  |  |  |  |  |
| 0 | 0 | 0 |  |  | 0 | 0 | 0,40 | 5,26 | 190,00 | 0 | 0 | 0 | 0 | 0 | 1 |
| 0 | 0 | 1 | 56,74 | 88,00 | 1 | 0 |  |  |  |  |  |  |  |  |  |
| 0 | 0 | 0 | 19,95 | 97,00 | 1 | 1 | 0,20 | 4,08 | 110,00 | 2 | 0 | 0 | 0 | 0 | 0 |
| 0 | 0 | 0 | 276,10 | 89,00 | 0 | 0 | 0,03 | 0,54 | 14,00 | 0 | 0 | 0 | 0 | 0 | 0 |
| 1 | 1 | 0 | 979,10 | 78,00 | 1 | 0 | 0,30 | 4,05 | 52,00 | 0 | 0 | 0 | 0 | 0 | 0 |
| 0 | 0 | 0 | 51,25 | 91,00 | 0 | 0 | 0,10 | 2,22 | 19,00 | 0 | 0 | 0 | 0 | 0 | 0 |
| 0 | 0 | 0 |  |  | 0 | 0 |  |  |  |  |  |  |  |  |  |
| 1 | 1 | 0 | 39,91 | 92,00 | 0 | 1 | 0,70 | 5,79 | 330,00 | 0 | 0 | 0 | 0 | 0 | 0 |
| 0 | 0 | 0 |  |  | 0 | 0 |  |  |  |  |  |  |  |  |  |
|  |  | 0 |  |  | 0 | 0 |  |  |  |  |  |  |  |  |  |
| 0 | 0 | 1 | 158,24 | 84,00 | 0 | 1 | 0,05 | 0,74 | 9,20 | 0 | 0 | 0 | 0 | 0 | 0 |
| 1 | 1 | 0 | 122,70 | 98,00 | 1 | 0 | 1,60 | 16,67 | 160,00 | 0 | 0 | 0 | 0 | 0 | 0 |
| 1 | 1 | 0 | 73,42 | 86,00 | 1 | 0 | 0,10 | 1,28 | 1200,00 | 0 | 3 | 6 | 0 | 0 | 1 |
| 1 | 1 | 0 | 30,70 | 83,00 | 1 | 0 | 0,20 | 2,60 | 200,00 | 0 | 0 | 0 | 0 | 0 | 0 |
| 0 | 0 | 0 | 22,86 | 83,00 | 1 | 0 | 0,04 | 0,83 | 12,00 | 0 | 0 | 0 | 0 | 0 | 0 |
| 1 | 1 | 0 | 121,34 | 85,00 | 1 | 0 | 0,40 | 8,00 | 210,00 | 0 | 0 | 0 | 0 | 0 | 1 |
| 0 | 0 | 0 | 56,51 | 94,00 | 0 | 1 | 0,40 | 4,17 | 97,00 | 4 | 0 | 4 | 0 | 0 | 1 |
| 1 | 1 | 0 | 35,77 | 95,00 | 1 | 1 | 0,20 | 2,99 | 390,00 | 0 | 4 | 0 | 0 | 0 | 1 |
| 0 |  | 0 |  |  | 0 | 0 |  |  |  |  |  |  |  |  |  |
| 0 | 0 | 1 |  |  | 0 | 0 |  |  |  |  |  |  |  |  |  |
| 0 | 0 | 0 |  |  | 0 | 0 |  |  |  |  |  |  |  |  |  |
| 1 | 1 | 0 | 251,81 | 89,00 | 1 | 0 | 0,07 | 1,09 | 32,00 | 0 | 0 | 0 | 0 | 0 | 0 |
| 0 | 0 | 0 |  |  | 0 | 0 |  |  |  |  |  |  |  |  |  |
| 0 | 0 | 0 |  |  | 1 | 1 |  |  |  |  |  |  |  |  |  |
| 0 | 0 | 0 | 55,91 | 82,00 | 1 | 0 | 0,20 | 2,53 | 37,00 | 0 | 0 | 0 | 0 | 0 | 0 |
| 1 | 1 | 0 |  | 92,00 | 0 | 0 | 0,40 | 8,00 | 47,00 | 0 | 0 | 0 | 0 | 0 | 0 |
| 0 | 0 | 0 |  |  | 1 | 0 |  |  |  |  |  |  |  |  |  |
| 0 | 1 | 0 |  |  | 1 | 0 |  |  |  |  |  |  |  |  |  |
| 0 | 1 | 0 | 110,00 | 79,00 | 0 | 0 | 0,09 | 1,48 | 20,00 | 0 | 2 | 0 | 0 | 0 | 0 |
| 1 | 1 | 1 | 32,20 | 83,00 | 0 | 1 | 0,90 | 6,92 | 45,00 | 0 | 0 | 0 | 0 | 0 | 0 |
| 1 | 1 | 0 | 244,90 | 59,00 |  |  | 0,60 | 8,70 | 330,00 | 4 | 7 | 9 | 6 | 5 | 1 |
| 1 | 1 | 0 | 115,76 | 89,00 | 0 | 1 | 0,20 | 2,74 | 18,00 | 0 | 0 | 4 | 0 | 0 | 1 |
|  |  | 0 |  |  | 1 | 0 |  |  |  |  |  |  |  |  |  |
| 0 | 1 | 0 |  |  | 0 | 0 |  |  |  |  |  |  |  |  |  |
| 1 | 1 | 0 | 26,50 | 89,00 | 0 | 1 | 0,20 | 4,26 | 570,00 | 5 | 6 | 0 | 1 | 0 | 1 |
| 1 | 1 | 0 | 4,84 | 91,00 | 1 | 0 | 0,30 | 3,23 | 420,00 | 4 | 7 | 0 | 0 | 0 | 1 |
| 0 | 0 | 0 | 38,40 | 81,00 | 0 | 1 |  |  |  |  |  |  |  |  |  |
| 1 | 1 | 0 | 70,45 | 78,00 | 0 | 1 | 0,20 | 2,35 | 100,00 | 1 | 6 | 0 | 0 | 0 | 1 |
| 0 | 0 | 0 | 25,03 | 87,00 | 0 | 1 | 0,20 | 2,74 | 56,00 | 3 | 0 | 0 | 0 | 0 | 1 |
| 0 | 0 | 1 | 872,72 | 86,00 | 0 | 0 | 0,20 | 2,78 | 11,00 | 0 | 0 | 0 | 0 | 0 | 0 |
| 0 | 0 | 0 | 23,49 | 84,00 | 1 | 0 | 0,10 | 1,27 | 31,00 | 0 | 0 | 0 | 0 | 0 | 0 |
| 0 | 0 | 0 | 59,43 | 88,00 | 0 | 0 | 0,09 | 1,20 | 70,00 | 0 | 0 | 0 | 0 | 0 | 0 |
| 0 | 0 | 0 | 110,00 | 84,00 | 0 | 1 | 0,30 | 5,08 | 26,00 | 0 | 0 | 0 | 0 | 0 | 0 |
| 1 | 1 | 0 |  |  | 0 | 1 |  |  |  |  |  |  |  |  |  |
| 1 | 1 | 1 |  |  | 0 | 0 |  |  |  |  |  |  |  |  |  |
| 0 | 0 | 0 | 20,09 | 96,00 | 0 | 0 | 0,70 | 7,61 | 210,00 | 0 | 0 | 0 | 0 | 0 | 0 |
| 0 | 0 | 0 | 115,03 | 87,00 | 1 | 0 | 0,10 | 1,47 | 4,70 | 0 | 0 | 0 | 0 | 0 | 0 |
| 0 | 0 | 2 | 2,60 | 94,00 | 1 | 0 | 0,20 | 4,17 | 5,70 | 0 | 0 | 0 | 0 | 0 | 0 |
| 0 | 0 | 0 | 17,16 | 87,00 | 0 | 1 | 0,20 | 2,67 | 13,00 | 0 | 0 | 0 | 0 | 0 | 0 |
| 0 | 0 | 0 |  |  | 0 | 0 |  |  |  |  |  |  |  |  |  |
| 0 | 0 | 1 | 120,50 | 85,22 | 0 | 0 | 0,40 | 5,33 | 530,00 | 0 | 0 | 0 | 0 | 0 | 0 |
| 0 | 0 | 1 |  |  | 0 | 0 |  |  |  |  |  |  |  |  |  |
| 0 | 0 | 1 | 24,64 | 92,37 | 0 | 0 | 0,20 | 4,26 | 18,00 | 0 | 0 | 0 | 0 | 0 | 0 |
| 0 | 0 | 0 | 47,30 | 81,22 | 0 | 1 | 0,30 | 4,92 | 63,00 | 0 | 0 | 0 | 0 | 0 | 0 |
| 0 | 0 | 2 |  | 87,61 | 0 | 0 | 0,20 | 2,50 | 42,00 | 0 | 0 | 0 | 0 | 0 | 0 |
| 0 | 0 | 2 |  |  | 0 | 1 |  |  |  |  |  |  |  |  |  |
| 0 | 0 | 2 |  |  | 0 | 0 |  |  |  |  |  |  |  |  |  |
| 1 | 1 | 1 | 1680,00 | 88,83 | 0 | 0 | 0,40 | 6,15 | 580,00 | 4 | 5 | 4 | 4,5 | 5 | 1 |
| 1 | 1 | 1 | 352,00 | 77,56 | 0 | 0 | 0,40 | 5,13 | 690,00 | 0 | 0 | 0 | 0 | 0 | 0 |
| 0 | 0 | 2 | 14,50 | 93,95 | 0 | 1 | 0,70 | 5,19 | 74,00 | 0 | 0 | 0 | 0 | 0 | 0 |
| 0 | 0 | 1 | 13,00 | 88,73 | 1 | 0 | 0,40 | 4,30 | 63,00 | 3 | 0 | 0 | 0 | 0 | 1 |
| 0 | 1 | 1 |  |  | 0 | 0 |  |  |  |  |  |  |  |  |  |
| 0 | 0 | 1 | 44,30 | 85,84 | 0 | 0 | 0,20 | 3,17 | 11,00 | 0 | 0 | 0 | 0 | 0 | 0 |
| 0 | 1 | 0 |  |  | 0 | 0 |  |  |  |  |  |  |  |  |  |
| 0 | 0 | 1 |  |  | 1 | 0 |  |  |  |  |  |  |  |  |  |
| 0 | 1 | 0 | 244,60 | 78,69 | 1 | 0 | 0,20 | 3,64 | 38,00 | 0 | 0 | 0 | 0 | 0 | 0 |
| 0 | 0 | 2 |  |  | 0 | 0 |  |  |  |  |  |  |  |  |  |
| 0 | 0 | 2 |  |  | 0 | 0 |  |  |  |  |  |  |  |  |  |
| 0 | 0 | 2 |  |  | 1 | 0 |  |  |  |  |  |  |  |  |  |
| 1 | 1 | 0 | 1656,00 | 89,07 | 0 | 0 | 0,40 | 6,45 | 200,00 | 0 | 0 | 0 | 0 | 0 | 1 |
| 0 | 0 | 2 |  |  | 0 | 0 |  |  |  |  |  |  |  |  |  |
| 1 | 1 | 0 | 22,70 | 92,54 | 0 | 0 | 0,10 | 1,19 | 180,00 | 0 | 6 | 0 | 0 | 0 | 1 |
| 0 | 1 | 0 | 240,00 | 99,32 | 0 | 1 | 0,40 | 5,19 | 130,00 | 0 | 0 | 0 | 0 | 0 | 0 |
| 1 | 1 | 0 | 55,90 | 85,13 | 1 | 1 | 0,90 | 13,24 | 140,00 | 0 | 0 | 0 | 0 | 0 | 0 |
| 0 | 1 | 2 |  |  | 1 | 0 |  |  |  |  |  |  |  |  | 0 |
| 0 | 0 | 2 | 104,00 | 86,48 | 0 | 0 | 0,20 | 2,82 | 9,80 | 0 | 0 | 0 | 0 | 0 |  |
| 1 | 1 | 2 |  |  | 1 | 0 |  |  |  |  |  |  |  |  | 0 |
| 0 | 1 | 1 | 218,29 | 83,78 | 1 | 0 | 0,70 | 11,11 |  | 0 | 0 | 0 | 0 | 0 |  |
| 0 | 1 | 0 |  |  | 0 | 1 |  |  |  |  |  |  |  |  | 0 |
| 0 | 0 | 1 | 2480,00 | 87,30 | 0 | 0 | 0,20 | 1,64 | 110,00 | 0 | 0 | 0 | 0 | 0 | 0 |
| 0 | 0 | 1 | 674,70 | 84,31 | 0 | 0 | 0,10 | 1,61 | 6,10 | 0 | 0 | 0 | 0 | 0 |  |
| 0 | 0 | 1 | 22,20 | 75,71 | 0 | 0 |  |  |  | 0 | 0 | 0 |  |  | 0 |
|  |  | 0 | 40,90 | 80,20 | 0 | 1 | 0,40 | 5,33 | 120,00 | 0 | 0 | 0 | 0 | 0 | 0 |
| 0 | 1 | 2 | 14,20 | 87,40 | 0 | 0 | 0,10 | 1,96 | 15,00 | 0 | 0 | 0 | 0 | 0 | 1 |
| 1 | 1 | 1 | 101,30 | 86,81 | 0 | 0 | 0,50 | 7,46 | 88,00 | 0 | 0 | 0 | 0 | 0 | 0 |
| 0 | 0 | 1 | 0,37 | 84,67 | 0 | 1 | 0,20 | 2,86 | 38,00 | 0 | 0 | 0 | 0 | 0 | 0 |
| 0 | 0 | 1 | 43,90 | 86,06 | 0 | 0 | 0,30 | 4,11 | 120,00 | 0 | 0 | 0 | 0 | 0 |  |
|  |  |  |  |  | 1 | 1 |  |  |  |  |  |  |  |  |  |
| 0 | 0 | 0 | 96,53 | 88,02 | 0 | 0 | 0,10 | 2,56 | 22,00 | 0 | 0 | 0 | 0 | 0 | 0 |
| 0 | 0 | 2 | 22,93 | 86,68 | 1 | 1 | 0,60 | 8,57 | 200,00 | 0 | 0 | 0 | 0 | 0 | 0 |
| 0 | 0 | 0 |  | 87,23 | 1 | 0 | 0,20 | 2,33 | 89,00 | 0 | 0 | 0 | 0 | 0 | 0 |
| 0 | 0 | 1 |  |  | 0 | 0 |  |  |  |  |  |  |  |  |  |
| 0 | 0 | 2 | 234,30 | 94,97 | 0 | 0 | 0,80 | 9,88 | 72,00 | 0 | 0 | 0 | 0 | 0 | 0 |
| 0 | 0 | 2 |  |  | 0 | 0 |  |  |  |  |  |  |  |  |  |
| 1 | 1 | 2 | 218,30 | 92,68 | 0 | 0 | 0,40 | 6,90 | 120,00 | 0 | 0 | 0 | 4 | 6 | 1 |
| 1 | 1 | 0 | 101,30 | 85,30 | 1 | 1 | 0,20 | 2,90 | 210,00 | 0 | 0 | 0 | 0 | 0 | 0 |
| 0 | 0 | 2 | 17,50 | 85,58 | 0 | 0 | 0,30 | 4,92 | 51,00 | 0 | 0 | 0 | 0 | 0 | 0 |
| 0 | 0 | 2 | 55,90 | 88,42 | 0 | 0 | 0,20 | 4,00 | 45,00 | 0 | 0 | 0 | 0 | 0 | 0 |
| 0 | 1 | 1 | 11,85 | 87,77 | 1 | 0 | 0,10 | 1,75 | 26,00 | 0 | 0 | 0 | 0 | 0 | 0 |
| 1 | 1 | 0 | 19,52 | 92,83 | 1 | 1 | 0,30 | 4,55 | 26,00 | 0 | 0 | 0 | 0 | 0 | 0 |
| 0 | 0 | 0 |  |  | 0 | 0 |  |  |  |  |  |  |  |  |  |
| 0 | 0 | 0 |  | 85,97 | 0 | 0 | 0,20 | 3,28 | 26,00 | 0 | 0 | 0 | 0 | 0 | 0 |
| 0 | 0 | 0 | 20,20 | 85,51 | 0 | 1 |  |  |  | 0 | 0 | 0 | 0 | 0 | 0 |
| 0 | 0 | 2 |  | 82,44 | 0 | 0 | 0,30 | 4,84 | 22,00 | 0 | 0 | 0 | 0 | 0 | 0 |
| 0 | 0 | 0 | 0,00 | 82,12 | 1 | 0 | 0,20 | 2,70 | 91,00 | 0 | 0 | 0 | 0 | 0 | 0 |
| 0 | 0 | 0 | 39,50 | 84,89 | 1 | 0 | 0,20 | 2,27 | 31,00 | 0 | 0 | 0 | 0 | 0 | 0 |
| 0 | 0 | 1 | 113,60 | 87,44 | 1 | 0 | 0,70 | 6,25 | 130,00 | 0 | 0 | 0 | 0 | 0 | 0 |
| 0 | 1 | 0 | 136,50 | 77,09 | 0 | 1 | 0,20 | 4,08 | 19,00 | 0 | 0 | 0 | 0 | 0 | 0 |
| 0 | 0 | 1 |  |  | 0 | 0 |  |  |  |  |  |  |  |  |  |
| 0 | 1 | 0 |  | 98,82 | 0 | 0 | 0,70 | 9,59 | 38,00 | 0 | 0 | 0 | 0 | 0 | 0 |
| 0 | 0 | 2 |  |  | 1 |  |  |  |  |  |  |  |  |  |  |
| 0 | 0 | 2 | 59,43 | 80,40 | 0 | 0 | 0,30 | 2,94 | 690,00 | 0 | 0 | 0 | 0 | 0 | 0 |
| 0 | 0 | 2 | 147,20 | 86,60 | 0 | 0 |  |  |  | 0 | 0 | 0 | 0 | 0 | 0 |
| 0 | 1 | 0 |  | 78,34 | 1 | 1 | 0,20 | 2,35 | 77,00 | 0 | 0 | 0 | 0 | 0 | 0 |
| 0 | 0 | 2 |  | 98,41 | 0 | 0 | 0,40 | 5,33 | 210,00 | 0 | 0 | 0 | 0 | 0 | 0 |
| 0 | 0 | 0 |  | 80,81 | 0 | 0 | 0,10 | 1,39 | 52,00 | 0 | 0 | 0 | 0 | 0 | 0 |
